# Supplementary material for: East Asian-specific and cross-ancestry genome-wide meta-analyses provide mechanistic insights into peptic ulcer disease
Source: Nat Genet. 2023 Nov 30;55(12):2129–38. doi: 10.1038/s41588-023-01569-7 (PMC10703676; doi:10.1038/s41588-023-01569-7)
Supplement: Supplementary file 1 — Supplementary Note and Figs. 1–39. [file 41588_2023_1569_MOESM1_ESM.pdf]

nature genetics

Article

https://doi.org/10.1038/s41588-023-01569-7

East Asian-specific and cross-ancestry genome-wide meta-analyses provide mechanistic insights into peptic ulcer disease

In the format provided by the authors and unedited

Supplementary materials

Table of Contents

Supplementary Note 2
Plausible pathways suggested by GWAS and pQTL analysis. 2
Genetic correlations between PUD and its risk factors 2
Potential roles of D cells and EC cells in PUD etiology 2
Comparison of fixed-effect and random-effects models in the meta-analysis of PUD in EAS 3
Comparison of GWASs using GRCh38-based datasets and GRCh37-based datasets 3
Supplementary methods 4
Cohort characteristics for BBJ1-180K, BBJ1-12K, BBJ2-42K ,and TMM-50K 4
Quality control for BBJ1-180K, BBJ1-12K, BBJ2-42K ,and TMM-50K 4
Cross-ancestry meta-analysis quality control 5
ABO blood group and secretor status interaction analysis 5
EAS LD reference panel construction for SBayesS 5
Supplementary Figures 6
Members of participating consortia 34
References 35

Supplementary Note

Plausible pathways suggested by GWAS and pQTL analysis.

By searching for pQTL associations[1–5], we found that the PUD risk alleles of lead variants at *ABO* and *GGT1* were linked with a reduced level of coagulation factor VIII (F8) and increased levels of factor X (*F10*) and PROS1(cofactor to activated protein C in the degradation of factor VIII). The risk allele of a lead SNP (rs1801020; *F12*) identified in the cross-ancestry meta-analysis is associated with decreased plasma levels of factor XII[6]. Factor XII, VIII, X, and PROS1 are all involved in the intrinsic pathway of blood coagulation[7,8]. These suggest that blood coagulation may be involved in peptic ulcer bleeding and healing. It is also likely that these signals were identified due to selection bias since PUD patients with severe symptoms are more likely to be detected than those without such severity. Additionally, in the pQTL study[1] that observed the proteomic associations with ABO blood groups and FUT2 secretor status, we found 31 proteins to be significantly associated with secretor status and non-O blood groups in the same direction (**Methods**). Notably, five proteins (CBLIF, CRNN, DSG2, REG1A, and REG1B) showed directionally concordant associations with secretor status and all three non-O blood groups (A, B, and AB) (**Supplementary Table 19**).

Genetic correlations between PUD and its risk factors

It has long been known cigarette smoking increases the risk for PUD[9]. In the genetic correlation analysis, we detected a nominally significant ($P<0.05$) negative correlation between PUD and age at smoking initiation, but such correlation was not detected for PUD and cigarettes per day, which suggest that PUD might share genetic components with long-term smoking behavior. We did not detect significant genetic correlations between PUD and drinking-related traits. The PUD risk allele for rs3859862 at *GGT1* (gamma-glutamyl transferase 1) identified in this study was linked with the alleles of a cis-eQTL and a cis-pQTL for *GGT1* that decrease the expression of *GGT1* in stomach and protein level of GGT in serum. It has been shown that alcohol consumption and smoking could lead to an increased level of GGT[10,11]. It is worth investigating if GGT plays a role in the association between PUD and smoking/drinking. Additionally, we identified nominally significant genetic correlations of PUD/GU with chronic obstructive pulmonary disease (COPD), asthma, and rheumatoid arthritis (RA) in EAS (**Supplementary Figure 12; Supplementary Table 21**). A previous large-scale meta-analysis of asthma[12] identified the significant genetic correlation between asthma and PUD in addition to the significant genetic correlation of asthma with COPD or RA. The consistent observations suggested a genetic link between PUD and immune-related diseases, which has not been well studied yet and warranted further investigations.

Potential roles of D cells and EC cells in PUD etiology

Gastrointestinal D cells are estimated to secret ~65% circulating somatostatin, suppressing the release of gastric hormones and gastric acid[13,14]. The PUD-associated rs2233580 in *PAX4* is a missense variant predicted to be highly deleterious (CADD > 20). It has been shown that PAX4 is a transcriptional repressor for somatostatin[15] and regulates duodenal hormone-secreting cells and serotonin/somatostatin-producing cells of the distal stomach[16,17]. We also detected significant associations of *PDX1* in gene-based tests with independent signals (rs139276646) in its regulatory region. PDX1 activates somatostatin transcription by interacting with its promoter[18]. These suggested that D cell/somatostatin dysregulation may contribute to PUD development. EC cells are the predominant source of body serotonin and play key roles in the gut-brain axis as chemosensors[19], affecting a wide range of physiological processes, including gastrointestinal motility and secretion, nausea, and visceral hypersensitivity. Psychological conditions, including stress and depression, were associated with a higher risk of PUD[20,21]. A previous large-scale study identified the

causal effect of major depression (MD) on PUD[22]. Given the important role of serotonin in psychological conditions, the association of EC cells/serotonin with PUD, and the bidirectional effects of the brain-gut axis, it is worth further investigating whether serotonin might be a key factor in the link between depression and PUD.

Comparison of fixed-effect and random-effects models in the meta-analysis of PUD in EAS

We conducted a random-effects meta-analysis using GWAMA[23] for PUD in EAS. 15 out of the 17 significant loci from the fixed-effect meta-analysis still showed significant association ($P < 5 \times 10^{-8}$) under the random-effects model (**Supplementary Figures 33**), with the other two loci being observed at the suggestive level ($P < 5 \times 10^{-6}$). We note that the analysis under the random-effects model was substantially underpowered ($\lambda_{GC} = 0.889$) compared with the fixed-effect model ($\lambda_{GC} = 1.052$). In the fixed-effect model, LD score regressions also supported no substantial inflation for the statistics obtained by the fixed-effect model ($\text{intercept}_{EAS,\ PUD} = 1.02$). Based on these results, we selected the fixed-effect approach for population-specific meta-analysis of PUD in this study.

Comparison of GWASs using GRCh38-based datasets and GRCh37-based datasets

To investigate the potential benefits of using imputation panels based on GRCh38/hg38, we processed and extracted the unrelated 2504 samples from the 1000 Genomes High Coverage datasets (GRCh38/hg38) as in the 1000 Genomes Project Phase3v5 panel (GRCh37/hg19). BBJ1-12K and BBJ2-42K were additionally imputed using the 1000 Genomes Project panel (GRCh38/hg38). We then conducted GWAS with the same settings and additional meta-analyses using the GRCh38-based datasets and GRCh37-based datasets from BBJ1-12K, BBJ2-42K, and FinnGen for comparison. The meta-analysis using GRCh38-based datasets showed overall high consistency of -log10(P) values ($r = 0.92$) with GRCh37-based results and consistent genomic inflation factors λ_{gc}, with substantially high consistency for significant variants ($r = 0.9991$). No additional novel loci were identified in GRCh38-specific regions. (**Supplementary Figures 34 -35**)

Supplementary methods

Cohort characteristics for BBJ1-180K, BBJ1-12K, BBJ2-42K ,and TMM-50K

BioBank Japan Project (BBJ, https://biobankjp.org/en/)[24], a hospital-based study, was founded in 2003, and enrolled approximately 200,000 participants of mainly Japanese ancestry, with at least one of 47 common diseases from 2003 to 2007, as the first cohort (BBJ1). From 2013 to 2017, BBJ additionally collected DNA and clinical information from 67,334 newly registered participants with at least one of 38 common diseases as the second cohort (BBJ2).

Genotype data of the case and control individuals included in the discovery-stage GWAS were obtained from the primary dataset of BBJ1, including 181,927 individuals (denoted as BBJ1-180K). Clinical information, including age and sex, was obtained from clinical records of BBJ-participating hospitals. In this study, we included samples of age ≥ 18 years. We performed principal component analysis (PCA) using 1000 Genomes Project[25] samples and then projected all samples onto the same space. We excluded outliers from the East Asian cluster.

Replication was conducted in three Japanese studies, namely an additional and independent set of BBJ1 cohort of 11,715 individuals (denoted as BBJ1-12K in this study, which was not included in the BBJ1-180K), a cohort of 42,689 individuals from BBJ2 (denoted as BBJ2-42K), and a population-based cohort of 49,621 individuals from Tohoku University Tohoku Medical Megabank (TMM; https://www.megabank.tohoku.ac.jp/english/) Project (denoted as TMM-50K)[26].

Quality control for BBJ1-180K, BBJ1-12K, BBJ2-42K ,and TMM-50K

All samples included in the discovery-stage GWAS (i.e., BBJ1-180K) were genotyped with either Illumina HumanOmniExpressExome BeadChips or a combination of Illumina HumanOmniExpress and HumanExome BeadChips. Samples from the BBJ1-12K and BBJ2-42K cohorts were genotyped with Infinium Asian Screening Array BeadChips. Samples from the TMM-50K cohort were genotyped with Axiom Japonica Array JPAv2 (Thermo Fisher Scientific, MA, USA). In the BBJ1-180K dataset, samples with call rates < 98% were excluded. We confirmed no sample in BBJ1-180K had excess heterozygosity (4 standard deviations (SD) from the mean). QC of autosomal genotypes was performed as described previously[62]. Briefly, we excluded the genotyped variants based on the following criteria for BBJ1-180K: (1) call rate < 99%, (2) heterozygote count < 5, (3) Hardy–Weinberg-equilibrium $P < 1.0 \times 10^{-6}$, and (4) concordance rate < 99.5% or non-reference discordance rate ≥ 0.5% between array genotypes and whole-genome-sequence dataset using overlapping participants (N = 939), as described previously[27]. We applied the same sample and variant QC criteria (except (4) for autosomal genotype QC) as in the discovery stage to the replication sets of BBJ1-12K and BBJ2-42K. Additionally, we removed samples with extreme heterozygosity rate (+/- 4 SD from the mean) in BBJ1-12K and BBJ2-42K, and the samples with amyotrophic lateral sclerosis in BBJ1-12K due to its comparatively high proportion. Non-random missingness in cases and controls was tested in BBJ1-180K, BBJ1-12K, and BBJ2-42K **(Supplementary Table 42).** In the replication using TMM-50K, we excluded samples with call rate < 95% and variants with (1) call rate < 99%, (2) heterozygote count < 5, or (3) Hardy–Weinberg-equilibrium $P < 1.0 \times 10^{-6}$. For QC of variants on chromosome X, we excluded genotyped variants with (1) call rate < 99% in males, females, or both, and (2) Hardy–Weinberg-equilibrium $P < 1.0 \times 10^{-6}$ in females.

Cross-ancestry meta-analysis quality control

Only autosomal variants in the 1KGp3v5 dataset were included in the meta-analysis; all variants were normalized[28], duplicate and multiallelic variants were removed for each dataset, and variants with imputation quality scores less than 0.3 were removed. For summary statistics from UKB-SAIGE (imputation quality scores not available), variants included in the previously published GWAS of PUD in UKB were kept, missing information of chromosome and base pair positions were assigned according to rsID, variants with extreme effect size values (|log(OR)| > 10) were removed, variants with minor allele count (MAC) < 5 were removed, and the strand of palindromic variants with MAF < 0.40 was further inferred using the allele frequencies obtained from each population in 1KGp3v5 dataset. Finally, we compared the effect allele frequencies in summary statistics and the population-specific alternative allele frequencies in 1KGp3v5. Variants with deviation in allele frequencies > 0.16 were excluded. In total, more than 19 million variants were included in the meta-analysis.

ABO blood group and secretor status interaction analysis

Logistic regression analyses adjusting for age, sex, and top 10 PCs were performed to examine the association of blood group or secretor status with PUD or subtypes. Blood group-specific effect sizes were estimated using the target blood group as exposure and combination of the other three blood groups as non-exposure (for example, individuals with A vs. B, AB, and O). Secretor status effect sizes were estimated considering the non-secretor status as exposure and the secretor status as non-exposure. To investigate the interaction of blood group O with non-secretor status, we further performed logistic regression analyses for blood group O–secretor interaction, adjusting for O blood group, FUT2 secretor status, age, sex, and top 10 PCs. Similarly, the blood group O – non-secretor status interaction was tested using imputed dosages of the variants determining O antigen and secretor status. All the above-mentioned logistic regressions were conducted in R v4.1.0. Additionally, we explored a previous pQTL study that had investigated proteomic associations with ABO blood groups and FUT2 secretor status for proteins associated with secretor status and A, B, and AB blood groups.

EAS LD reference panel construction for SBayesS

Briefly, a full LD matrix on HapMap3 SNPs was computed using 50,000 randomly selected and unrelated East Asian individuals from BBJ1-180K. The off-diagonal entries of full LD matrix were shrunk with the interpolated genetic map for the 1000 Genomes Project JPT population (https://github.com/joepickrell/1000-genomes-genetic-maps). The effective population size and genetic map sample size were set to 11,600 and 100, respectively, according to 1000 Genomes Project phased OMNI data (http://ftp.1000genomes.ebi.ac.uk/vol1/ftp/technical/working/20130507_omni_recombination_rates/). The sparse shrunk LD matrix used for SBayesS was created by setting elements of the shrunk matrix to zero if their chi-squared statistic under the sampling distribution of the correlation coefficient did not exceed 10.

The MHC region was excluded. We ran four parallel MCMC chains with a length of 50,000 and a burn-in size of 20,000 for each trait. To evaluate the convergence in MCMC, potential scale reduction statistics for each parameter were computed. Traits with potential scale reduction statistic < 1.2 for all three parameters, including SNP-based heritability, polygenicity estimates, and S, were considered to have good convergence, and were, therefore, used in the study.

Supplementary Figures

Table of contents

Supplementary Figure 1. Study workflow. 8
Supplementary Figure 2. Venn diagram of cases used in the discovery-stage GWAS. 8
Supplementary Figure 3. Manhattan plots and Q-Q plots for PUD and its subtypes from the discovery-stage GWAS in BBJ1-180K. 9
Supplementary Figure 4. Manhattan plots and Q-Q plots for PUD and its subtypes from the East Asian-specific meta-analysis. 9
Supplementary Figure 5. Manhattan plots and Q-Q plots for PUD and its subtypes from the cross-ancestry meta-analysis. 10
Supplementary Figure 6. Phenogram of genome-wide significant loci for PUD and its subtypes. 11
Supplementary Figure 7. Effect size comparison of lead variants with and without the winner's curse corrections. 12
Supplementary Figure 8. Cross-ancestry effect size comparison of lead variants for PUD and its subtypes. 13
Supplementary Figure 9. Overlap between PUD signals and significant cis-eQTL variants of the GTEx database. 14
Supplementary Figure 10. Genetic correlation heatmap of PUD and dietary habits in East Asian population. 15
Supplementary Figure 11. Genetic correlation heatmap of PUD and quantitative traits in East Asian population. 15
Supplementary Figure 12. Genetic correlation heatmap of PUD and binary traits in East Asian population. 15
Supplementary Figure 13. PheWAS heatmap of PUD risk variants with ATC codes and quantitative traits. 16
Supplementary Figure 14. PheWAS heatmap of PUD risk variants with binary traits (Part 1/2). 17
Supplementary Figure 15. PheWAS heatmap of PUD risk variants with binary traits (Part 2/2). 18
Supplementary Figure 16. Summary of significant associations identified in PheWAS. 19
Supplementary Figure 17. Effect size comparison of distinct signals for *H.pylori*-stratified analysis in East Asian ancestry individuals. 19
Supplementary Figure 18. Effect size comparison of distinct signals for DU and GU in East Asian ancestry individuals. 20
Supplementary Figure 19. Effect size comparison of distinct signals for DUonly and GUonly in BBJ1-180K. 21
Supplementary Figure 20. Effect size comparison of distinct signals for DU and GU in European ancestry individuals. 21
Supplementary Figure 21. Cross-cohort effect size comparison of distinct signals for DU and GU in East Asian ancestry individuals. 22
Supplementary Figure 22. Polygenicity estimation in East Asians using SBayesS. 23
Supplementary Figure 23. Posterior distribution of polygenicity estimates for GU and DU in EAS using SbayesS. 23
Supplementary Figure 24. Statistical power estimation for Mendelian randomization analysis. 24
Supplementary Figure 25. Effect size comparison of distinct signals between PUD and GC in East Asian ancestry individuals. 24
Supplementary Figure 26. Cell-type specificity analysis in East Asian ancestry individuals using LDSC. 25
Supplementary Figure 27. Cell-type specificity analysis in East Asian ancestry individuals using MAGMA. 25
Supplementary Figure 28. Cell-type specificity analysis in European ancestry individuals using LDSC. 26
Supplementary Figure 29. Cell-type specificity analysis in European ancestry individuals using MAGMA. 26
Supplementary Figure 30. Cross-ancestry meta-analysis of cell-type specificity using LDSC. 27
Supplementary Figure 31. Cross-ancestry meta-analysis of cell-type specificity using MAGMA. 27
Supplementary Figure 32. Venn Plot of the potential sample overlap within Biobank Japan cohorts. 28
Supplementary Figure 33. Manhattan plots and Q-Q plots for EAS-specific meta-analysis of PUD using different models. 29
Supplementary Figure 34. Manhattan plots and Q-Q plots for cross-ancestry meta-analyses based on different versions of reference genome. 30
Supplementary Figure 35. Comparison of -log10(P) for SNPs in GWASs based on different versions of reference genome. 30
Supplementary Figure 36. Power analysis for GWAS of HP-negative PUD in TMM-50K. 31
Supplementary Figure 37. Comparison of MAGMA results using different window sizes around genes for gene-based analysis. 32
Supplementary Figure 38. Comparison of MAGMA results using different window sizes around genes for tissue-specificity analysis. 33
Supplementary Figure 39. Comparison of MAGMA results using different window sizes around genes for cell-type-specificity analysis. 34

Abbreviations

PUD: peptic ulcer diseases
GU: gastric ulcers (including samples with both gastric ulcers and duodenal ulcers)
GUonly: gastric ulcers only (excluding samples with both gastric ulcers and duodenal ulcers
DU: duodenal ulcers (including samples with both gastric ulcers and duodenal ulcers)
DUonly: duodenal ulcers only (excluding samples with both gastric ulcers and duodenal ulcers)
BU: comorbidity of gastric ulcers and duodenal ulcers
GC: gastric cancers
HP: *Helicobacter pylori*
BBJ1: Biobank Japan 1st cohort
BBJ2: Biobank Japan 2nd cohort
BBJ1-180K: 180,000 individuals from BBJ1 (the main BBJ1 dataset)
BBJ1-12K: additional 12,000 individuals from BBJ1 (not included in the main BBJ1-180K dataset)
BBJ2-42K: 42,000 individuals from BBJ2
TMM-50K: 50,000 individuals from Tohoku Medical Megabank Project
UKB: UK Biobank
QC: quality control
GWAS: genome-wide association study
PheWAS: phenome-wide association analysis
Q-Q : quantile-quantile
λ_{gc}: genomic inflation factor
eQTL: expression quantitative trait loci
pQTL: protein quantitative trait loci
ATC: Anatomical Therapeutic Chemical code
EAS: East Asian
EUR: European
LDSC: linkage disequilibrium score regression

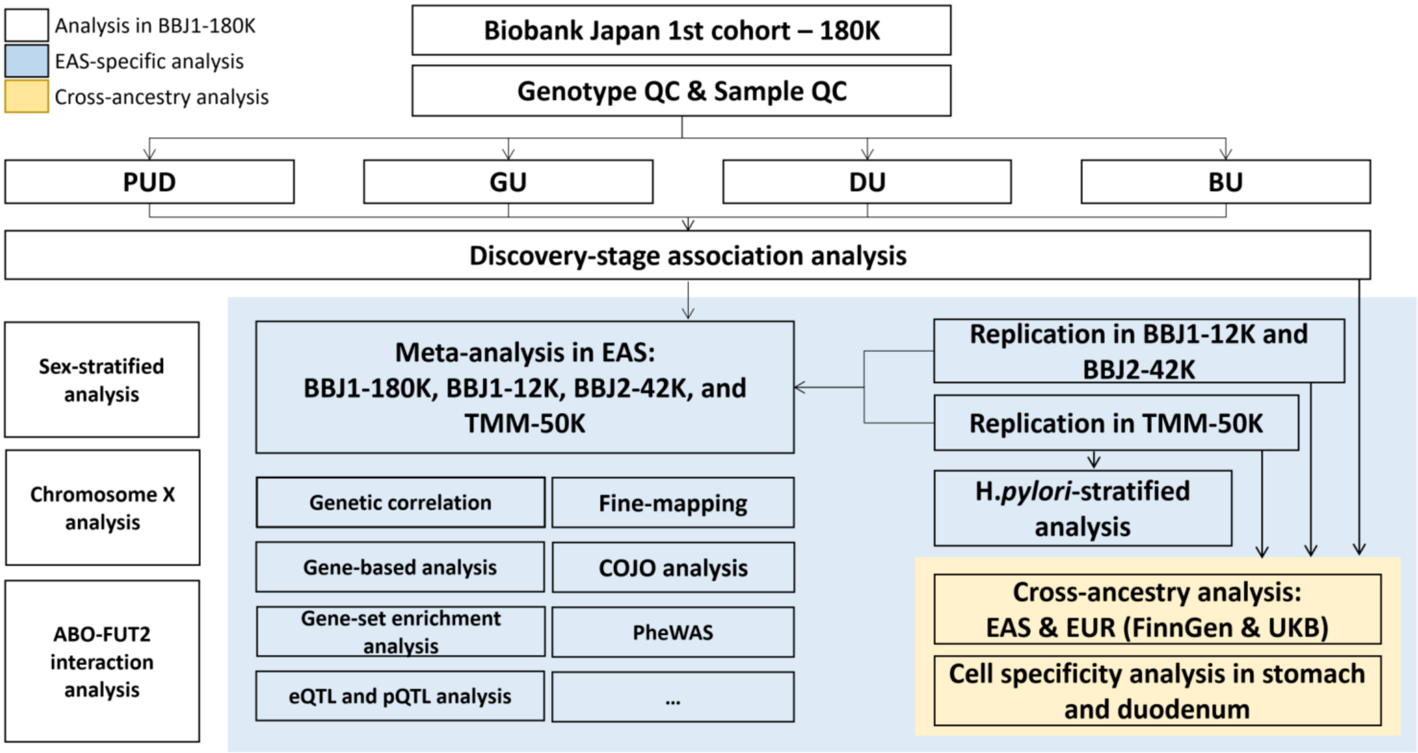

Supplementary Figure 1. Study workflow.

The three-stage design of this study was shown. We first performed discovery-stage GWAS for PUD and its subtypes in BBJ1-180K and then conducted replication in three independent studies. Next, East Asian-specific meta-analyses were conducted for PUD and its subtypes combining the four studies, and post-GWAS analyses were performed mainly using East Asian-specific summary statistics. Finally, we carried out cross-ancestry meta-analyses for PUD, DU, and GU, combining the four East Asian studies and GWASs in FinnGen and UKB.

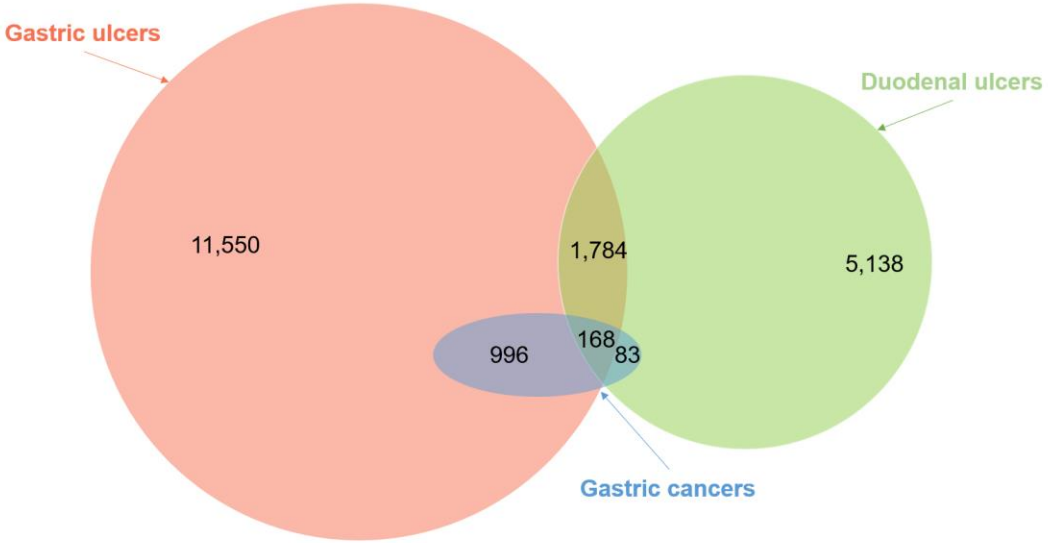

Supplementary Figure 2. Venn diagram of cases used in the discovery-stage GWAS.

Phenotype overlap among individuals with gastric ulcers, duodenal ulcers, and gastric cancers was shown for PUD cases in BBJ1-180K.

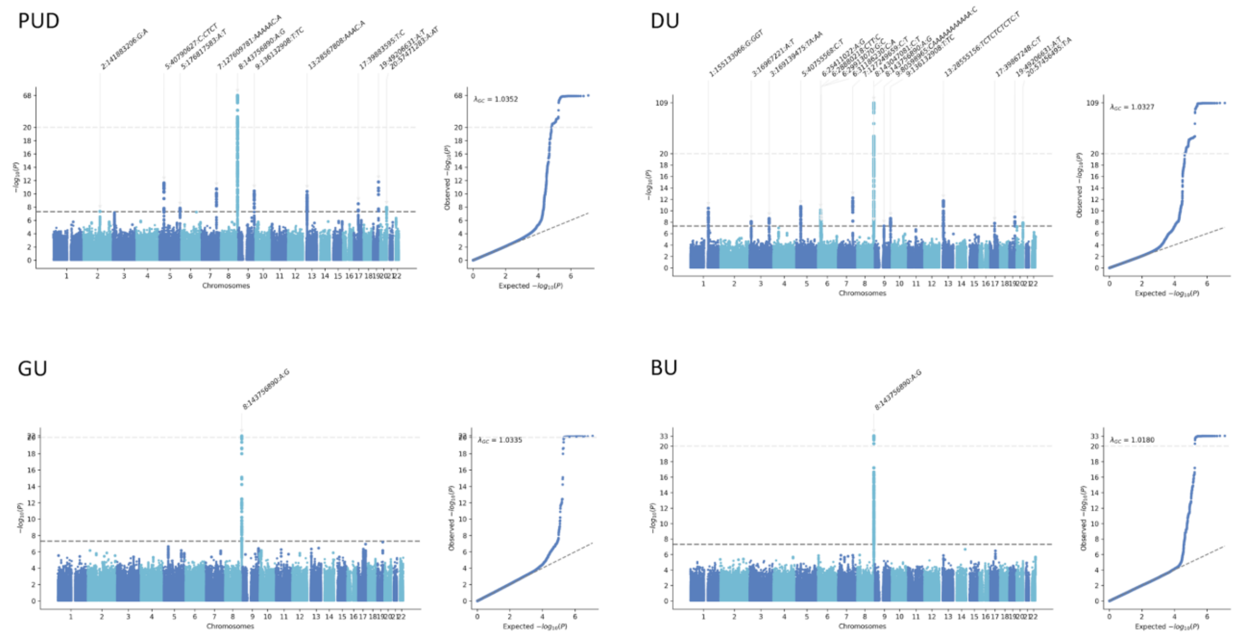

Supplementary Figure 3. Manhattan plots and Q-Q plots for PUD and its subtypes from the discovery-stage GWAS in BBJ1-180K.

Two-sided P values were derived from the discovery-stage GWAS in BBJ1-180K. Variants are plotted against GRCh37. For variants above the top light grey dashed line ($-\log_{10}(P) > 20$), P values are rescaled. Significant loci are annotated with the lead variant. The bottom dark grey dashed line indicates the genome-wide significance threshold ($P < 5.0 \times 10^{-8}$).

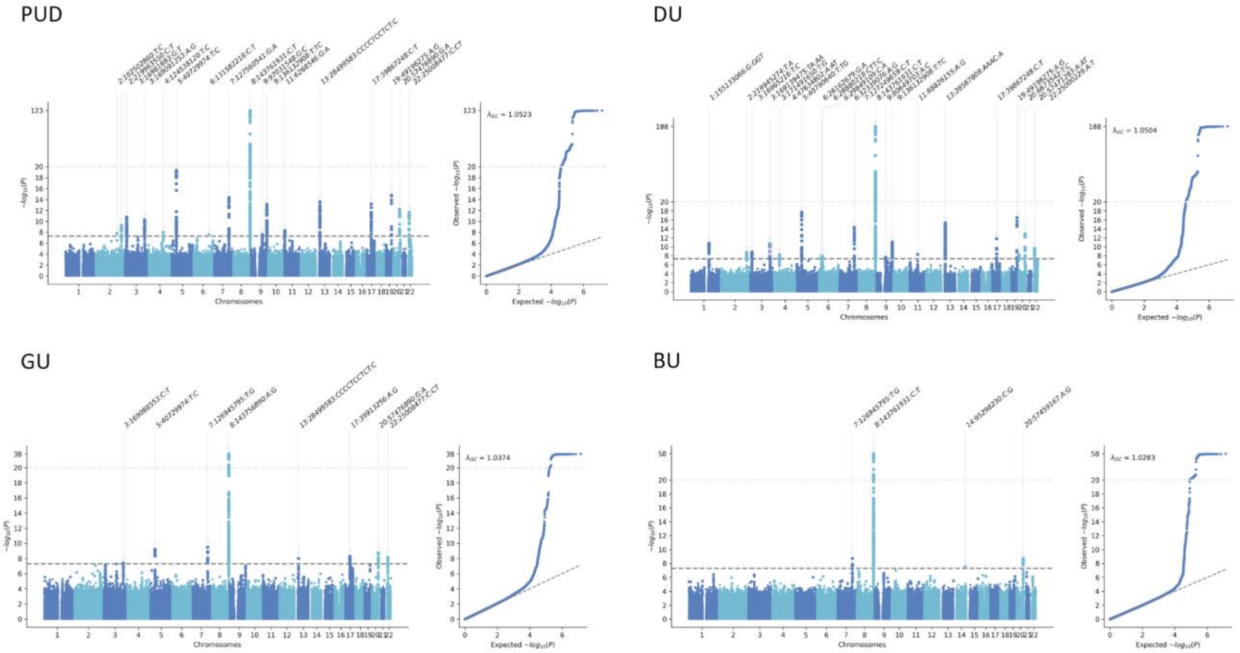

Supplementary Figure 4. Manhattan plots and Q-Q plots for PUD and its subtypes from the East Asian-specific meta-analysis.

Two-sided P values were derived from the East Asian-specific meta-analyses combining the four studies. Variants are plotted against GRCh37. For variants above the top light grey dashed line($-\log_{10}(P) > 20$), P values are rescaled. Significant loci are annotated with the lead variant. The bottom dark grey dashed line indicates the genome-wide significance threshold ($P < 5.0 \times 10^{-8}$).

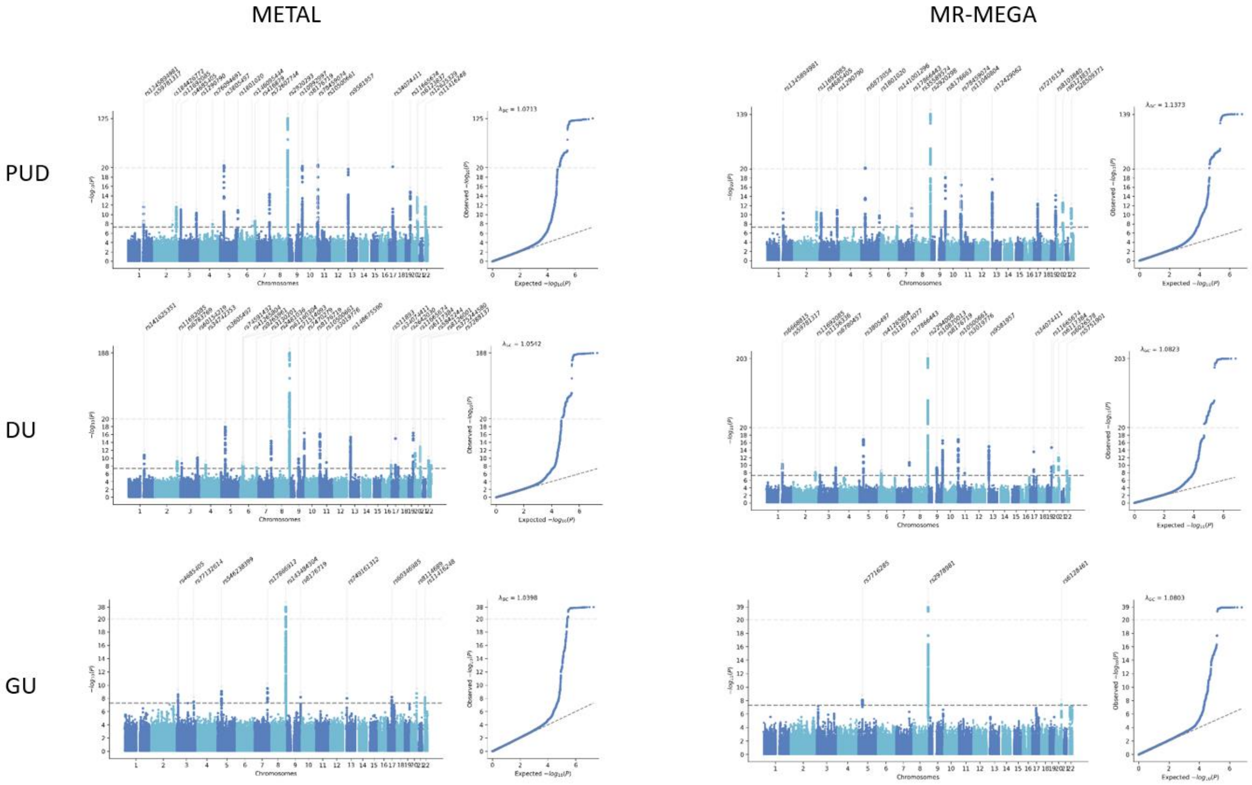

Supplementary Figure 5. Manhattan plots and Q-Q plots for PUD and its subtypes from the cross-ancestry meta-analysis.

Two-sided P values were derived from the cross-ancestry meta-analyses using either METAL or MR-MEGA. For summary statistics from MR-MEGA, P values were recalculated from Chi-square statistics. Variants are plotted against GRCh37. For variants above the top light grey dashed line ($-\log_{10}(P) > 20$), P values are rescaled. Significant loci were annotated with the lead variant. The bottom dark grey dashed line indicates the genome-wide significance threshold ($P < 5.0 \times 10^{-8}$).

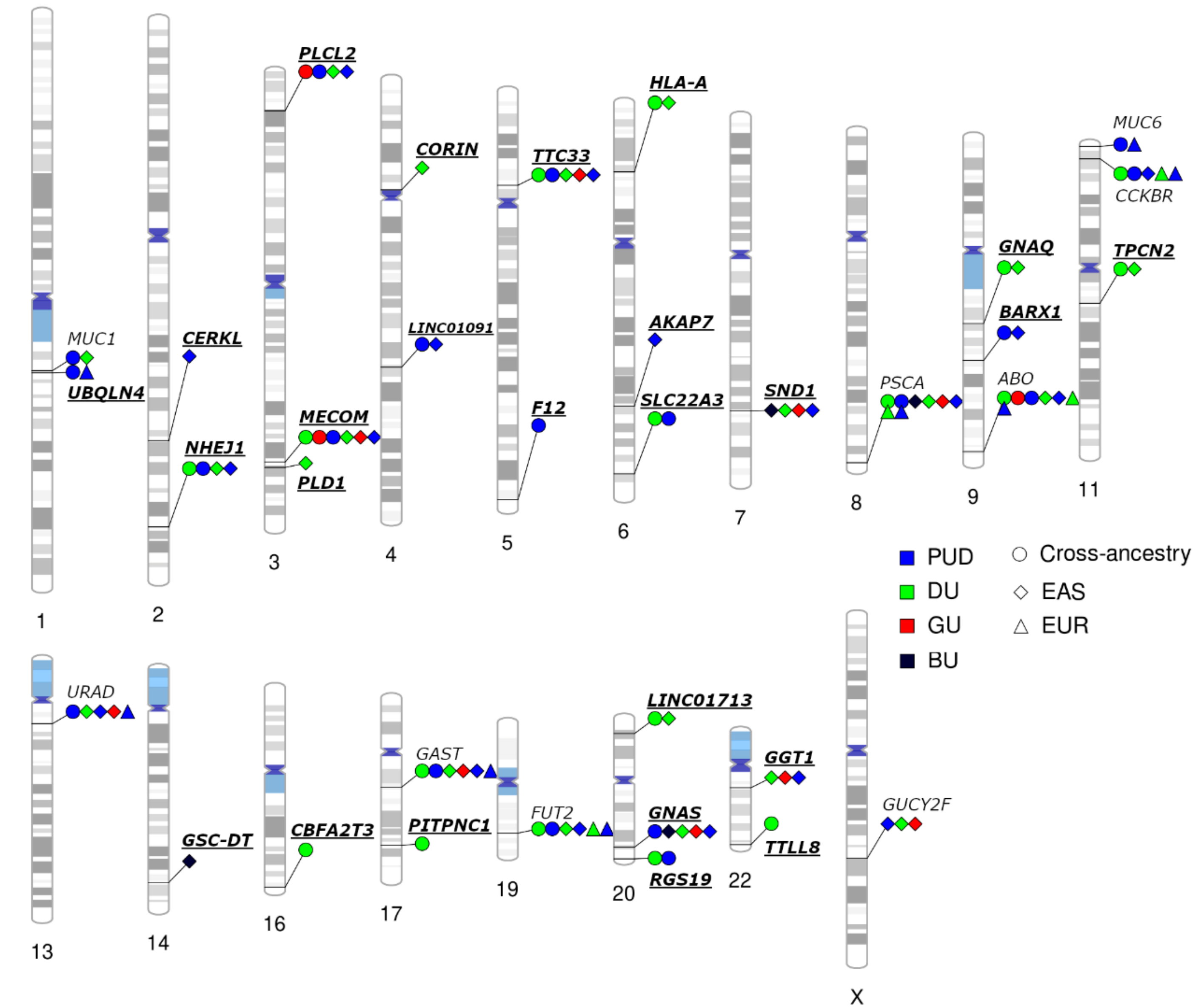

Supplementary Figure 6. Phenogram of genome-wide significant loci for PUD and its subtypes.

The shapes indicate ancestry: circles, cross-ancestry (Cross-ancestry); diamonds, East Asians (EAS); triangles, Europeans (EUR). Colors indicate peptic ulcer disease (PUD) and its subtypes: blue, PUD; green, Duodenal ulcers (DU); red, Gastric Ulcers (GU); black, Both gastric and duodenal ulcers (BU). Identified loci are annotated with the nearest genes to the most significant lead variants. Novel loci were indicated by bold and underlined gene names. Cytobands and annotations are based on GRCh37/hg19. Chromosomes with no associated loci are omitted.

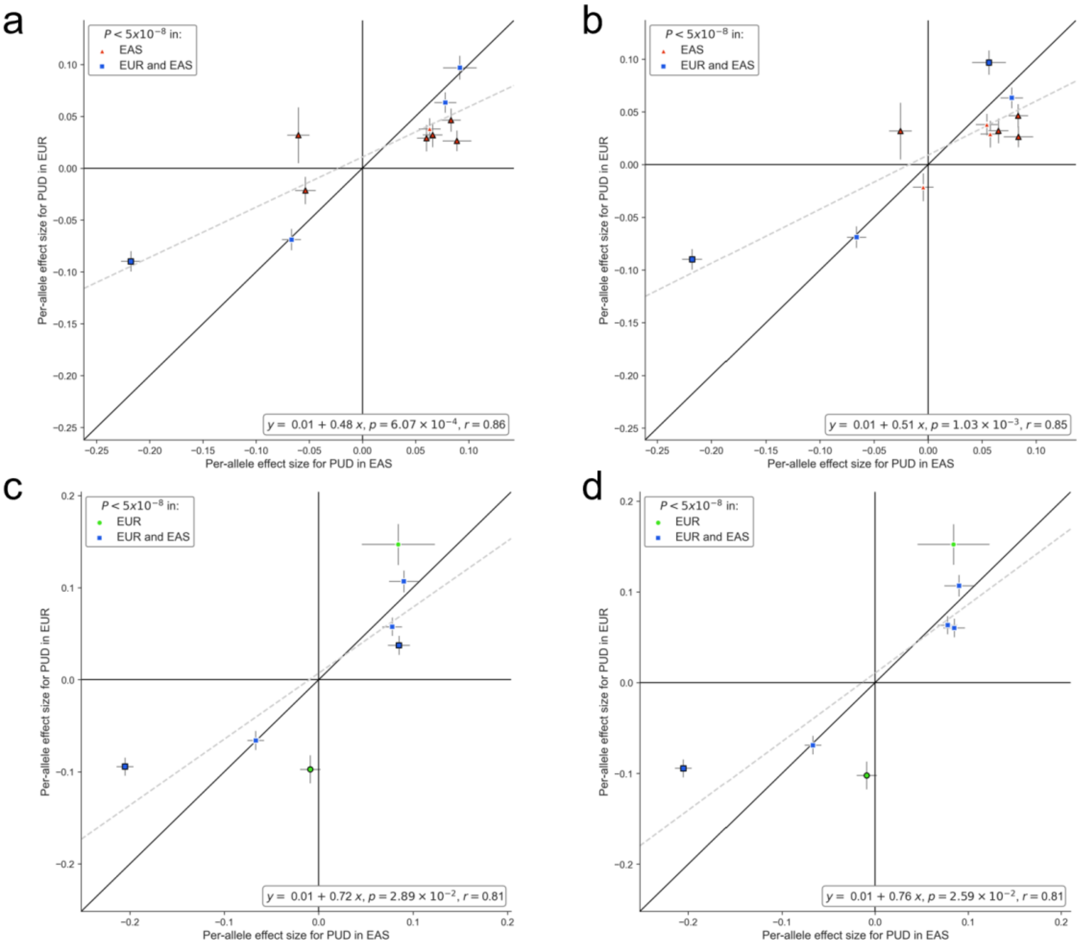

Supplementary Figure 7. Effect size comparison of lead variants with and without the winner's curse corrections.

Data are presented as effect size estimates (log(OR)) +/- standard errors. Two-sided P values were derived from the ancestry-specific meta-analyses using METAL. The grey dashed line represents the fitted linear regression line with annotation at the bottom right (P values are derived from two-sided t-tests for the slopes). **a,** comparison using lead variants of significant loci ascertained in EAS. **b**, the winner's curse-corrected comparison using lead variants of significant loci ascertained in EAS. **c**, comparison using lead variants of significant loci ascertained in EUR. **d**, the winner's curse-corrected comparison using lead variants of significant loci ascertained in EUR. Source data are provided in Supplementary Table 11.

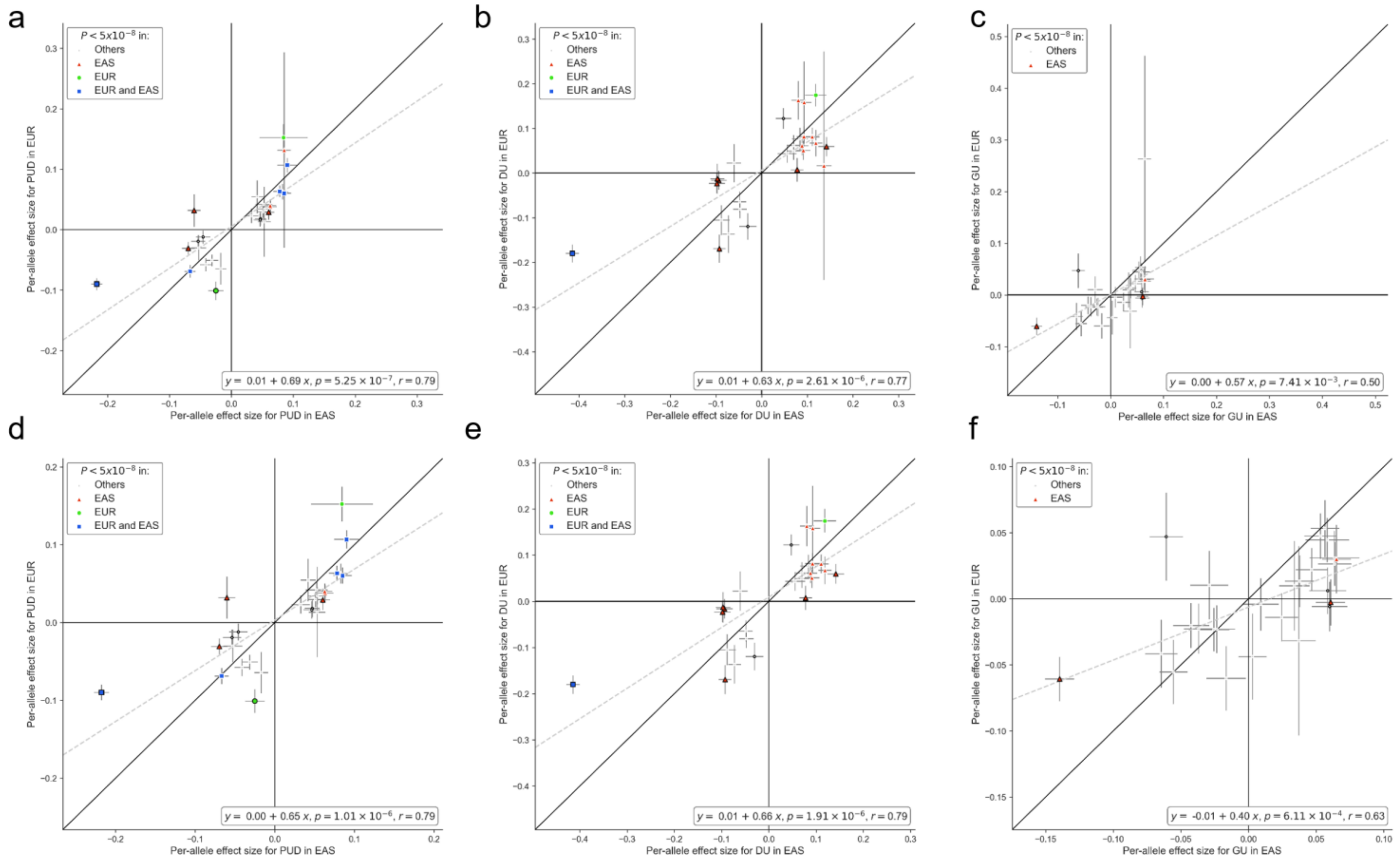

Supplementary Figure 8. Cross-ancestry effect size comparison of lead variants for PUD and its subtypes.

Per-allele effect size (log(OR)) comparison using EAS-specific and EUR-specific summary statistics for PUD and its subtypes. Lead variants associated with PUD or any subtypes in EAS-specific, EUR-specific, or cross-ancestry meta-analysis were selected for comparison (GWAS P values are two-sided). The most significant associations are shown if overlapping variants exist (interval < 500 kb). Data are presented as effect size estimates (log(OR)) +/- standard errors. Variants with significant heterogeneity (Cochran's Q test; two-sided $P_{het} < 0.05$) are denoted by the black marker edges. The grey dashed line represents the fitted linear regression line with annotation at the bottom right (P values are derived from two-sided t-tests for the slopes). **a**, 28 available variants (existing in both datasets). **b**, 27 available variants. **c**, 27 available variants. **d**, 27 available variants with MAF > 0.01. **e**, 26 available variants with MAF > 0.01. **f**, 26 available variants with MAF > 0.01. Source data are provided in Supplementary Table 10.

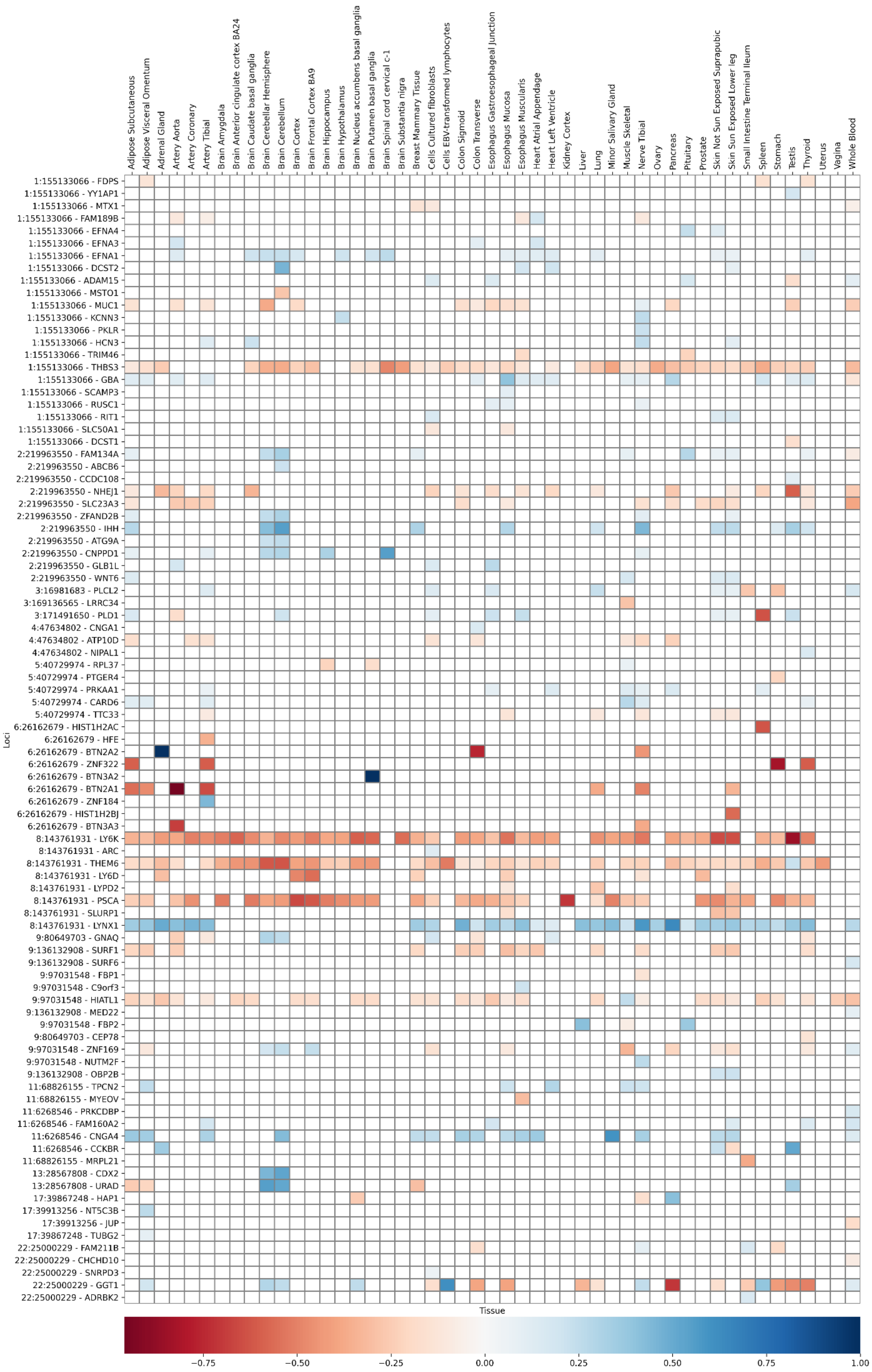

Supplementary Figure 9. Overlap between PUD signals and significant cis-eQTL variants of the GTEx database.

Overlap of significant cis-eQTL variants (FDR < 5%) of the GTEx version 8 datasets[29] with lead variants in novel loci or its LD proxy ($r^2 > 0.6$ in 1KG EAS or EUR populations[25]) in 41 tissue types. Square colors represent the normalized beta values (slope of the linear regression in eQTL mapping) of the eQTL allele that is in LD with the PUD risk allele. For each transcript, only the most significant eQTL association is shown. Columns represent the tissue types, and rows show the genome coordinates of PUD lead variants (GRCh37) and target transcripts of the overlapping eQTL association.

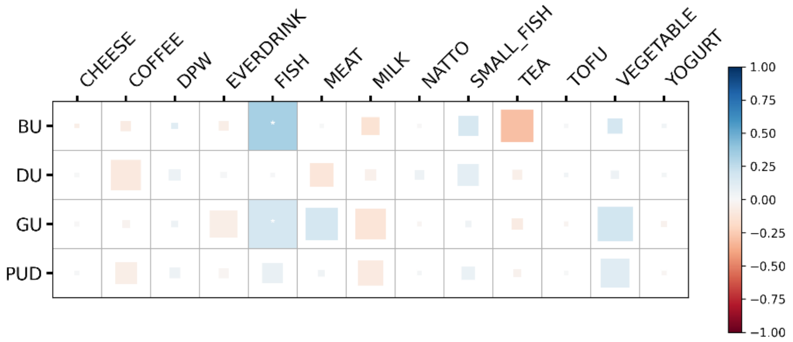

Supplementary Figure 10. Genetic correlation heatmap of PUD and dietary habits in East Asian population.

Genetic correlation among PUD and dietary habits in BBJ1-180K[30](**Methods**). None of the genetic correlations reached the significance threshold (FDR < 5%) after corrections. The size of the colored square in each cell is proportional to the $-\log_{10}(P)$ (two-sided P values) of its corresponding correlation. *, P < 0.05. Source data are provided in Supplementary Table 21.

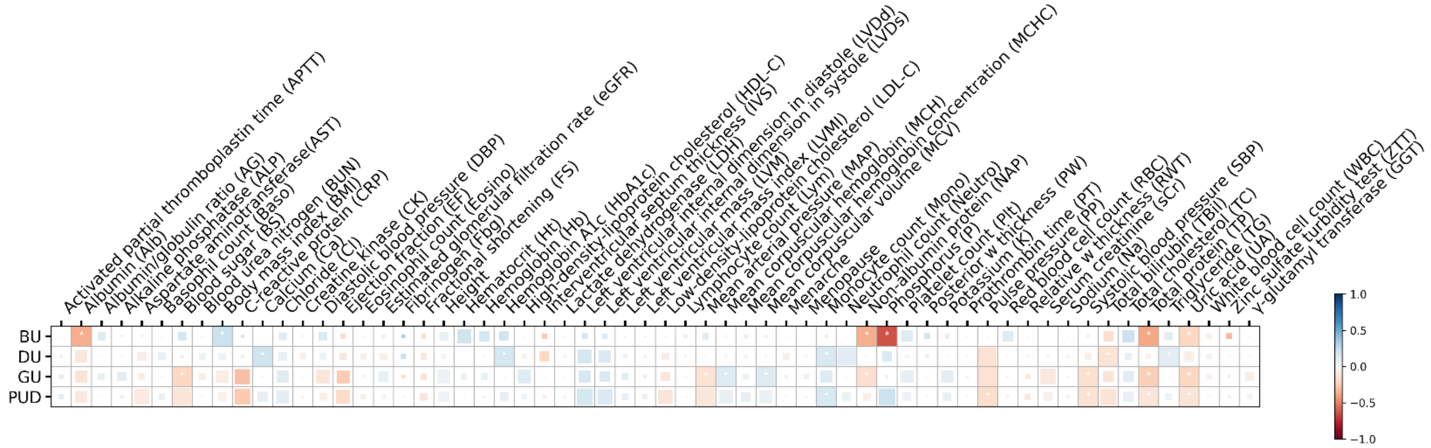

Supplementary Figure 11. Genetic correlation heatmap of PUD and quantitative traits in East Asian population.

Genetic correlation among PUD and quantitative traits in BBJ1-180K[31] (**Methods**). None of the genetic correlations reached the significance threshold (FDR < 5%) after corrections. The size of the colored square in each cell is proportional to the $-\log_{10}(P)$ (two-sided P values) of its corresponding correlation. *, P < 0.05. Source data are provided in Supplementary Table 21.

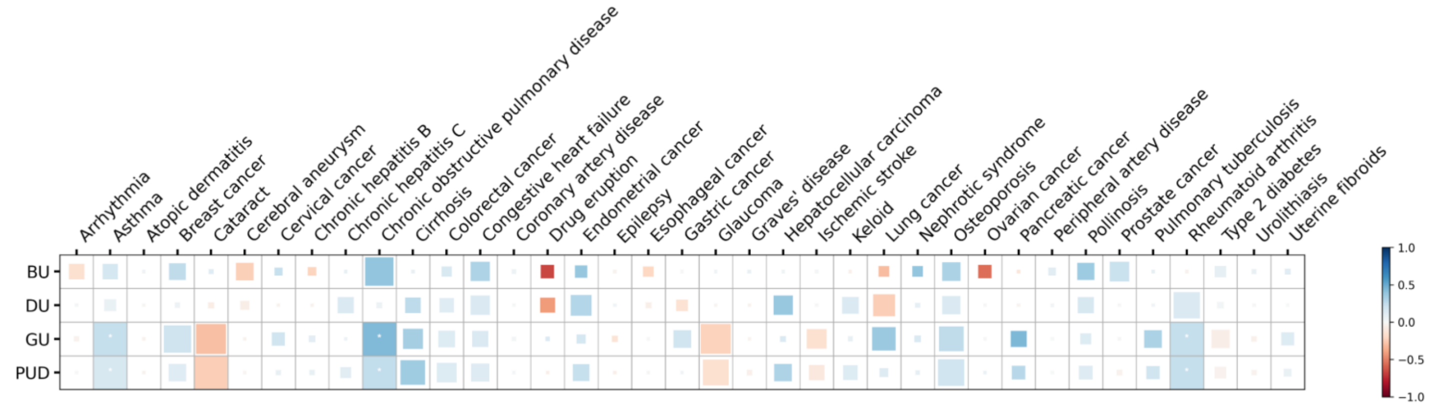

Supplementary Figure 12. Genetic correlation heatmap of PUD and binary traits in East Asian population.

Genetic correlation among PUD and binary traits in BBJ1-180K[32] (**Methods**). None of the genetic correlations reached the significance threshold (FDR < 5%) after corrections. The size of the colored square in each cell is proportional to the $-\log_{10}(P)$ (two-sided P values) of its corresponding correlation. *, P < 0.05. Source data are provided in Supplementary Table 21.

.

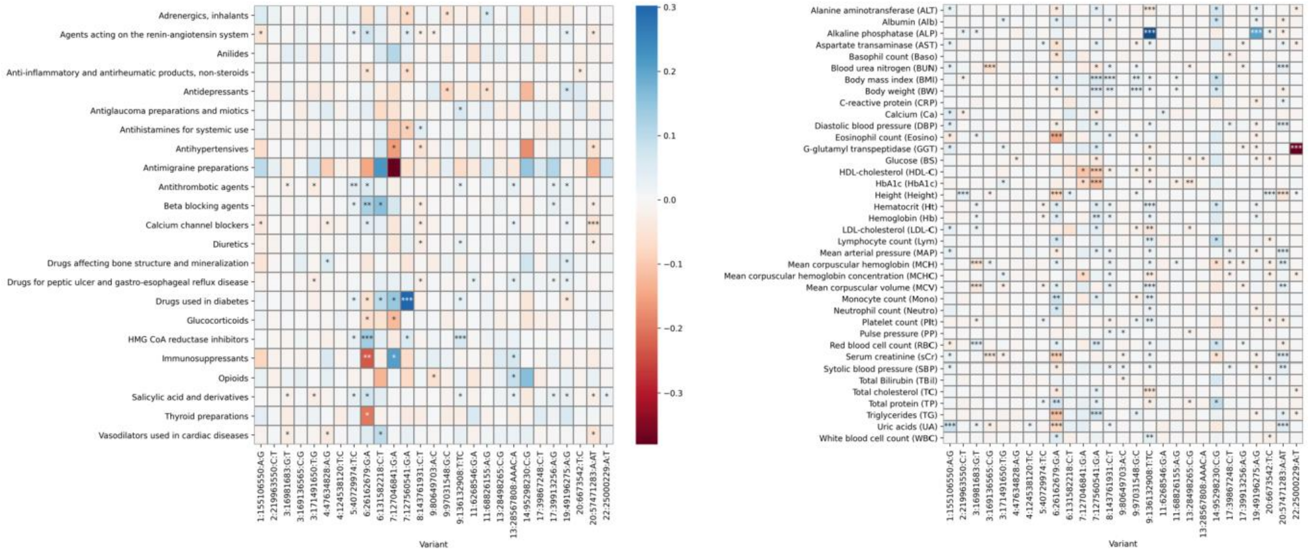

Supplementary Figure 13. PheWAS heatmap of PUD risk variants with ATC codes and quantitative traits.

Lead variants and independent secondary signals associated with PUD or any subtypes in the EAS population were selected for PheWAS lookup. a, ATC codes. b, quantitative traits. The most significant associations were selected if overlapping variants exist (interval < 500 kb). Summary statistics for ATC codes and quantitative traits were obtained from previous GWASs in BBJ1[33]. Per-allele effect sizes (log(OR)) of the PUD risk alleles are shown. *, two-sided $P < 0.05$ (Nominal significance). **, two-sided $P < 8.6 \times 10^{-6}$ (Bonferroni correction). ***, two-sided $P < 5.0 \times 10^{-8}$ (Genome-wide significance). Source data are provided in Supplementary Table 22.

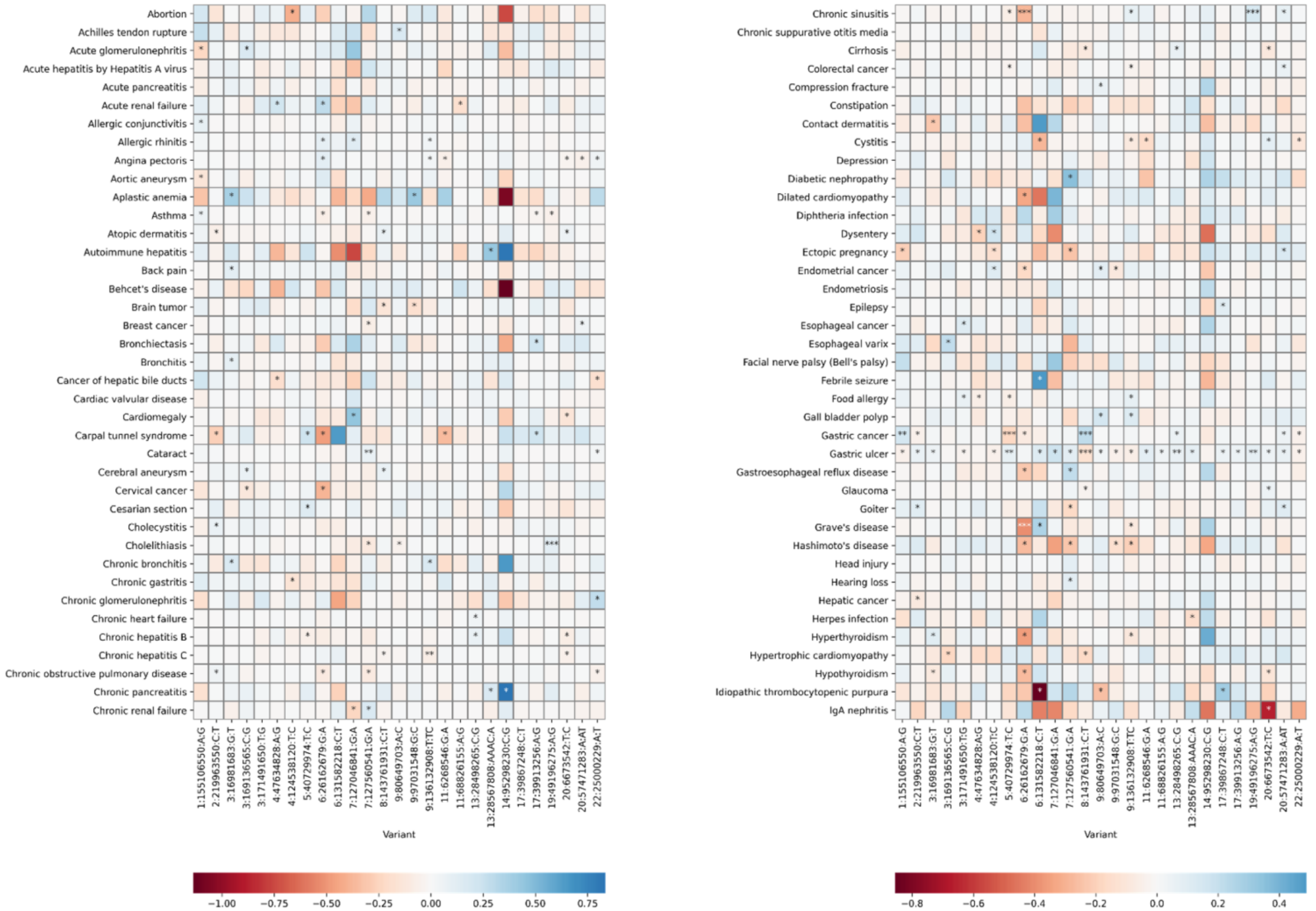

Supplementary Figure 14. PheWAS heatmap of PUD risk variants with binary traits (Part 1/2).

Lead variants and independent secondary signals associated with PUD or any subtypes in the EAS population were selected for PheWAS lookup. The most significant associations were selected if overlapping variants exist (interval < 500 kb). Summary statistics for binary traits were obtained from previous GWASs in BBJ1-180K[33]. Per-allele effect sizes (log(OR)) of the PUD risk alleles are shown. *, two-sided $P < 0.05$ (Nominal significance). **, two-sided $P < 8.6 \times 10^{-6}$ (Bonferroni correction). ***, two-sided $P < 5.0 \times 10^{-8}$ (Genome-wide significance). Source data are provided in Supplementary Table 22.

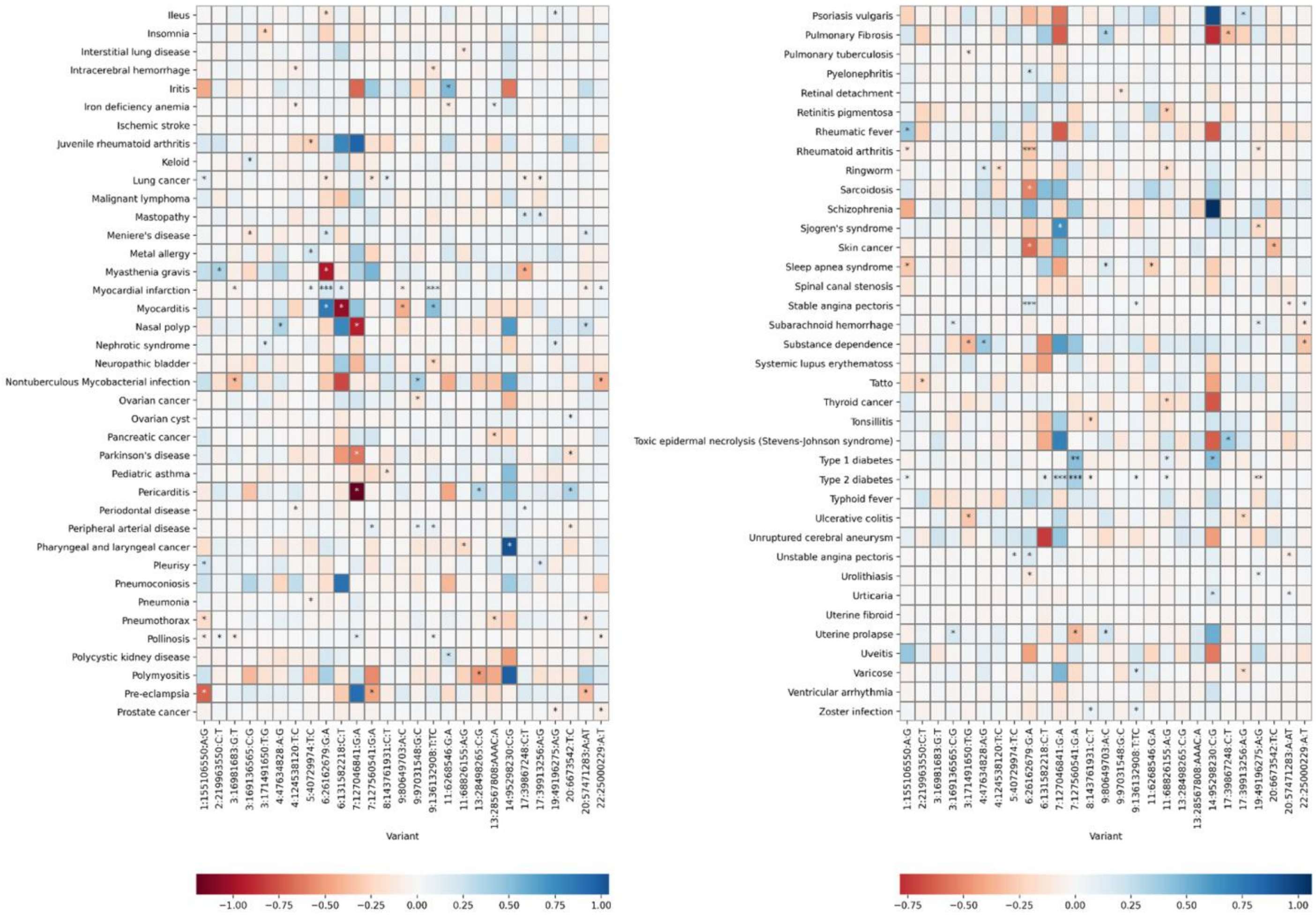

Supplementary Figure 15. PheWAS heatmap of PUD risk variants with binary traits (Part 2/2).

Lead variants and independent secondary signals associated with PUD or any subtypes in the EAS population were selected for PheWAS lookup. The most significant associations were selected if overlapping variants exist (interval < 500 kb). Summary statistics for binary traits were obtained from previous GWASs in BBJ1-180K[33]. Per-allele effect sizes (log(OR)) of the PUD risk alleles are shown. *, two-sided P < 0.05 (Nominal significance). **, two-sided $P < 8.6 \times 10^{-6}$ (Bonferroni correction). ***, two-sided $P < 5.0 \times 10^{-8}$ (Genome-wide significance). Source data are provided in Supplementary Table 22.

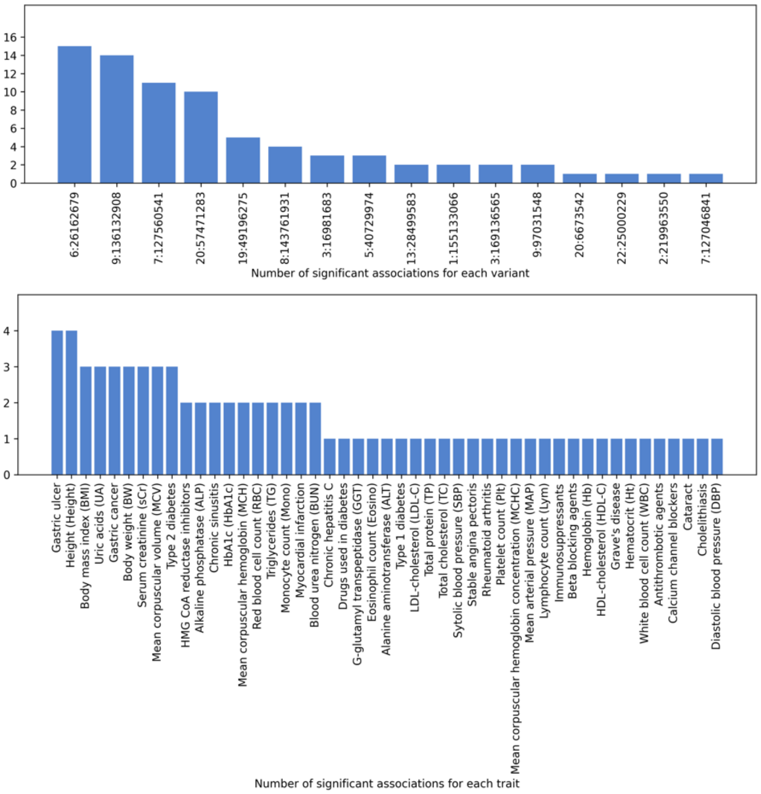

Supplementary Figure 16. Summary of significant associations identified in PheWAS.

Genome-wide significant associations identified in PheWAS lookup are summarized for each variant (top) or each trait (bottom).

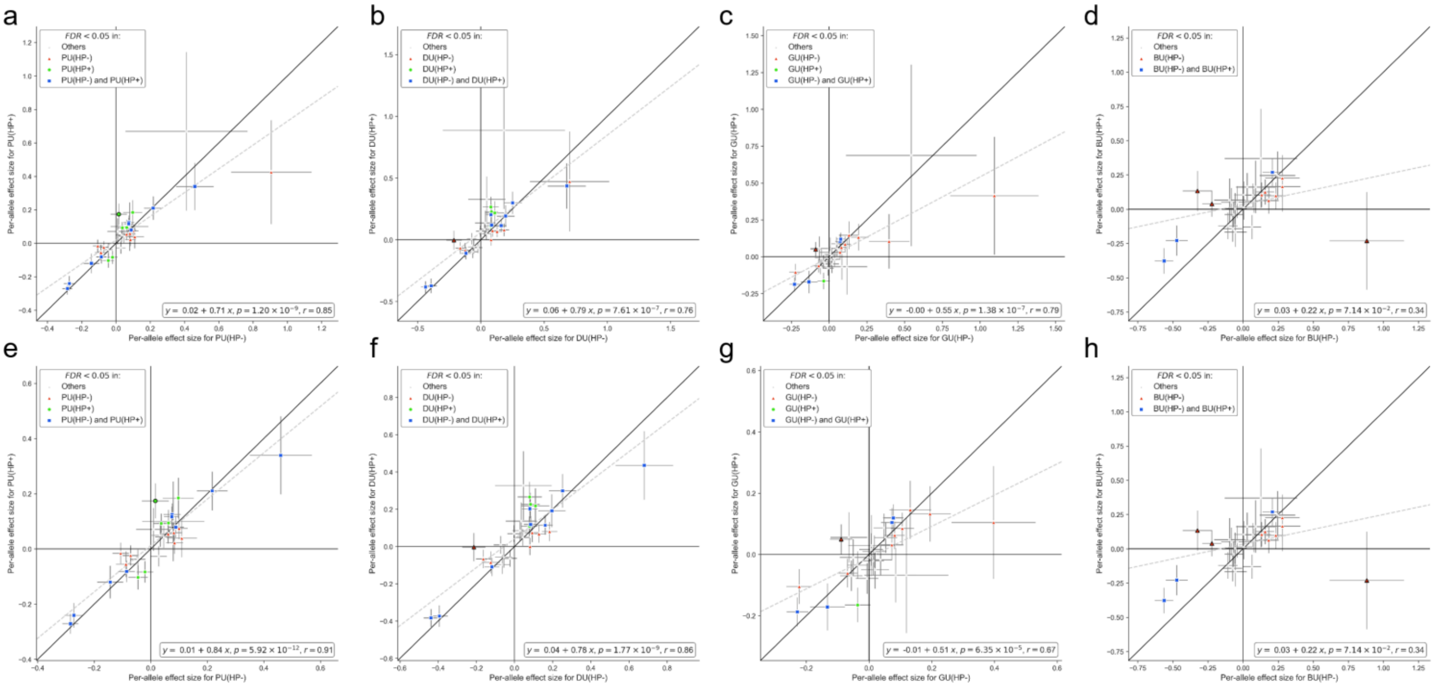

Supplementary Figure 17. Effect size comparison of distinct signals for *H.pylori*-stratified analysis in East Asian ancestry individuals.

Per-allele effect size (log(OR)) comparison using summary statistics from *H.pylori*-stratified analysis. PUD (HP+), *H.pylori*-positive PUD; PUD (HP-), *H.pylori*-negative PUD; GU (HP+), *H.pylori*-positive GU; GU (HP-), *H.pylori*-negative GU; DU (HP+), *H.pylori*-positive DU; DU (HP-), *H.pylori*-negative DU; BU (HP+), *H.pylori*-positive BU; BU (HP-), *H.pylori*-negative BU. Lead variants and

independent secondary signals associated with PUD or any subtypes in the EAS population were selected for comparison (GWAS P values are two-sided). The most significant associations are shown if overlapping variants exist (interval < 500 kb). Data are presented as effect size estimates (log(OR)) +/- standard errors. The grey dashed line represents the fitted linear regression line with annotation at the bottom right (P values are derived from two-sided t-tests for the slopes). **a, e,** PUD. **b, f,** GU. **e, g,** DU. **d, h,** BU. **a, b, c, d,** 31 available variants (existing in both datasets) are shown. **e, f, g, h,** 29 available variants with MAF > 0.01 are shown. Source data are provided in Supplementary Table 24.

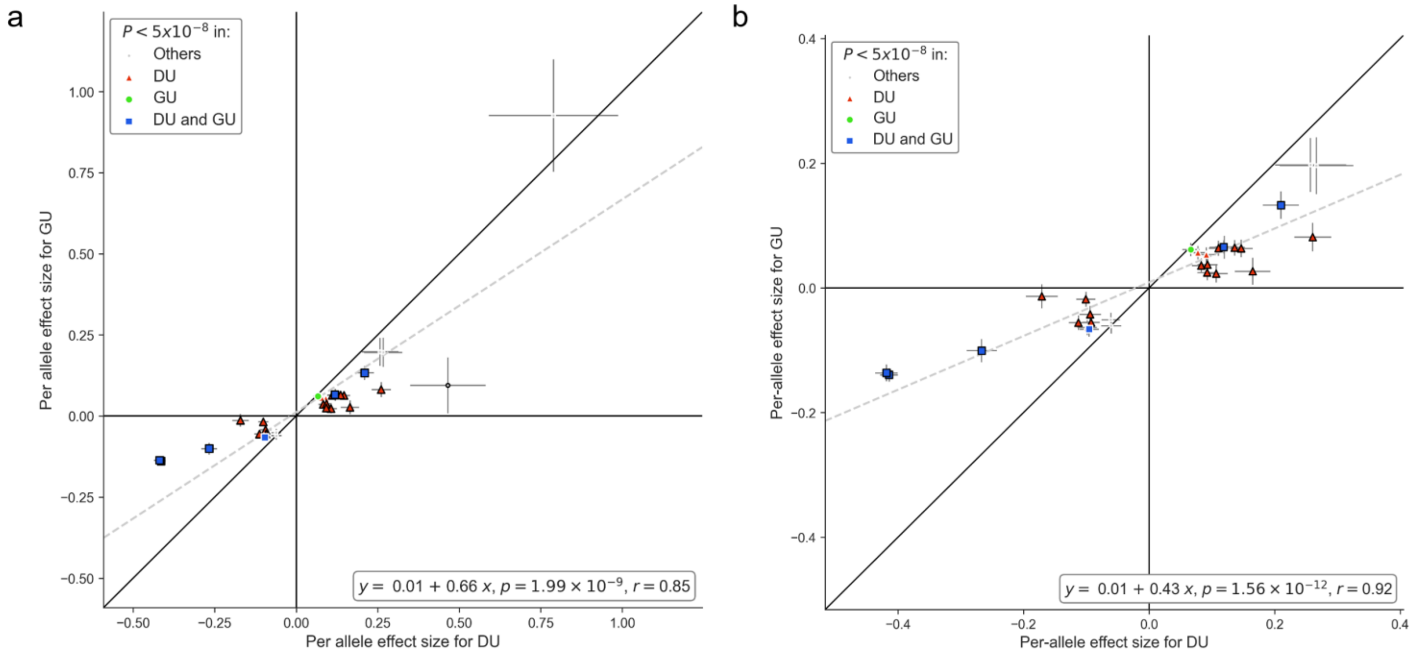

Supplementary Figure 18. Effect size comparison of distinct signals for DU and GU in East Asian ancestry individuals.

Per-allele effect size (log(OR)) comparison using EAS-specific summary statistics for DU and GU. Lead variants and independent secondary signals associated with PUD or any subtypes in the EAS population were selected for comparison (GWAS P values are two-sided). The most significant associations were shown if overlapping variants exist (interval < 500 kb). Data are presented as effect size estimates (log(OR)) +/- standard errors. Variants with nominal significant heterogeneity (Cochran's Q test; two-sided $P_{het} < 0.05$) were denoted by black marker edges. The grey dashed line represents the fitted linear regression line with annotation at the bottom right (P values are derived from two-sided t-tests for the slopes).. Marker colors denote the GWAS in which the significant variants are identified; Others, PUD or BU. **a,** all 31 available variants (existing in both datasets) are shown. **b,** 29 variants with MAF > 0.01 are shown. Source data are provided in Supplementary Table 26.

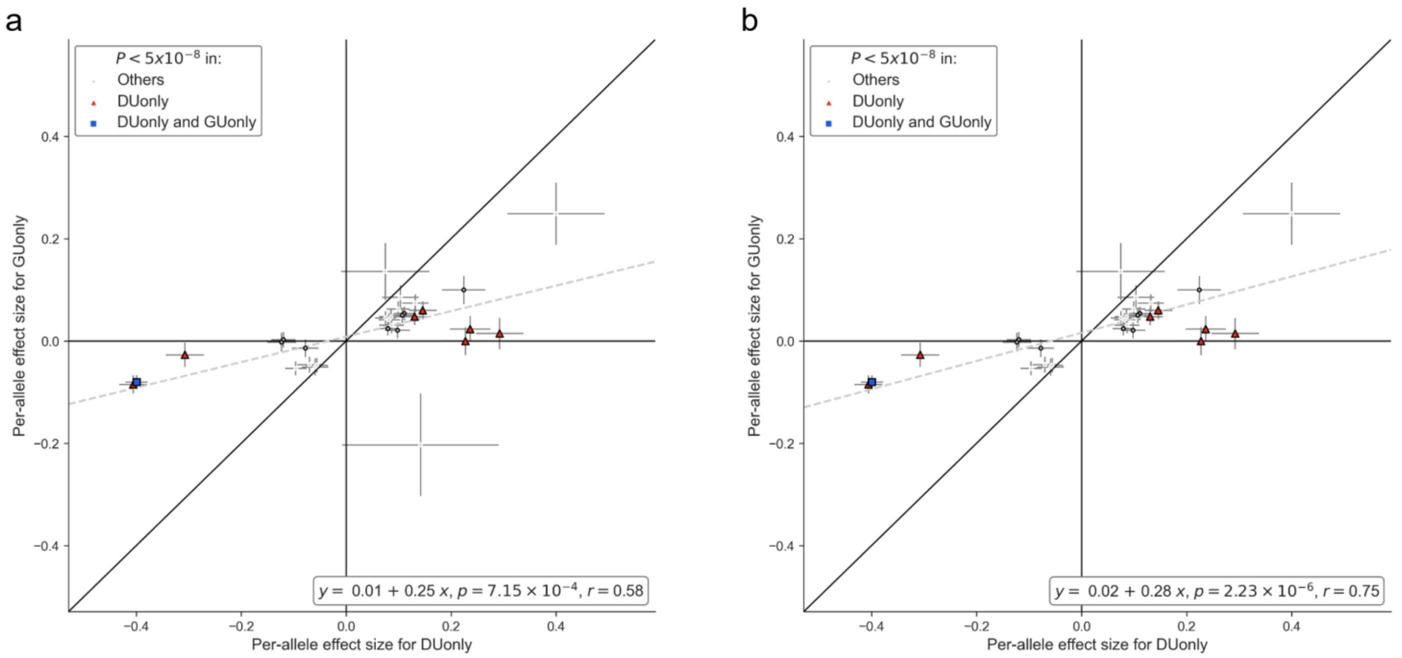

Supplementary Figure 19. Effect size comparison of distinct signals for DUonly and GUonly in BBJ1-180K.

Per-allele effect size (log(OR)) comparison using summary statistics for DUonly and GUonly in BBJ1-180K. Lead variants and independent secondary signals associated with PUD or any subtypes in the EAS population were selected for comparison (GWAS P values are two-sided). The most significant associations are shown if overlapping variants exist (interval < 500 kb). Data are presented as effect size estimates (log(OR)) +/- standard errors. Variants with significant heterogeneity (Cochran's Q test; two-sided $P_{het} < 0.05$) are denoted by black marker edges. The grey dashed line represents the fitted linear regression line with annotation at the bottom right (P values are derived from two-sided t-tests for the slopes). Marker colors denote the GWAS in which the significant variants were identified; Others, PUD or BU. **a.** 30 available variants (existing in both datasets) are shown. **b.** 29 available variants with MAF>0.01 are shown. Source data are provided in Supplementary Table 27.

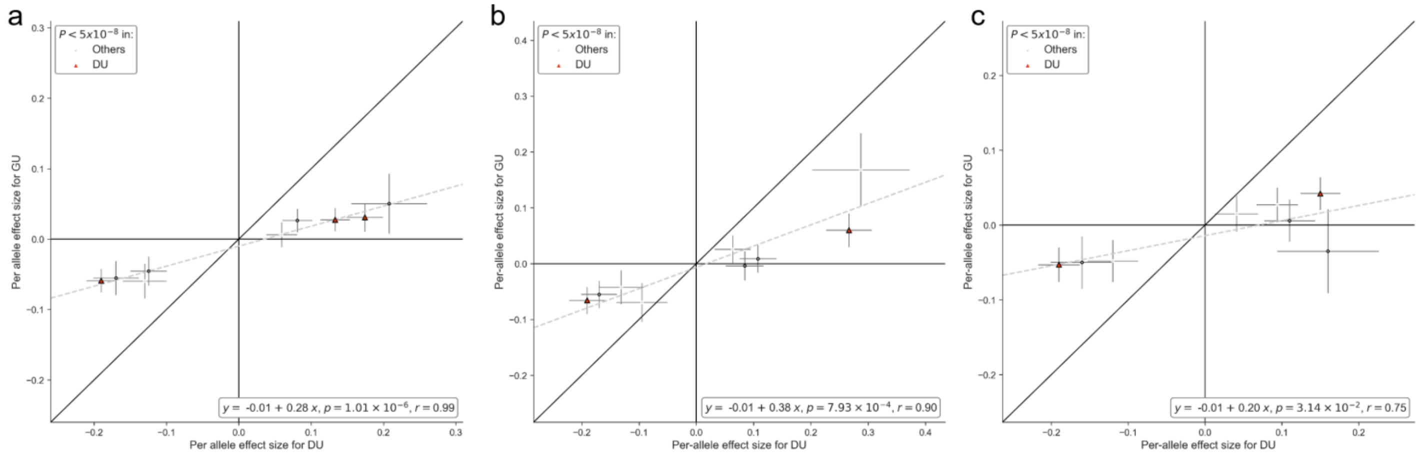

Supplementary Figure 20. Effect size comparison of distinct signals for DU and GU in European ancestry individuals.

Per-allele effect size (log(OR)) comparison using summary statistics for DU (N = 631,686) and GU (N = 634,243) derived from EUR-specific meta-analysis (**a**), GWASs in UKB (**b**), and GWASs in FinnGen (**c**). Details were described in **Methods**. Lead variants associated with PUD or any subtypes in the EUR population were selected for comparison (GWAS P values are two-sided). The most significant associations were shown if overlapping variants exist (interval < 500 kb). Data are presented as effect size estimates (log(OR)) +/- standard errors. Variants with significant heterogeneity (Cochran's Q test; two-sided $P_{het} < 0.05$) are denoted by black marker edges. The grey dashed line represents the fitted linear regression line with annotation at the bottom right (P values are derived from two-sided t-tests for the slopes). Marker colors denote the GWAS in which the significant variants were identified; Others, PUD. **a, b,** 9 available variants (existing in both datasets) are shown. **c,** 8 available variants are shown. Source data are provided in Supplementary Table 28. Sample sizes for UKB and FinnGen are provided in Supplementary Table 7.

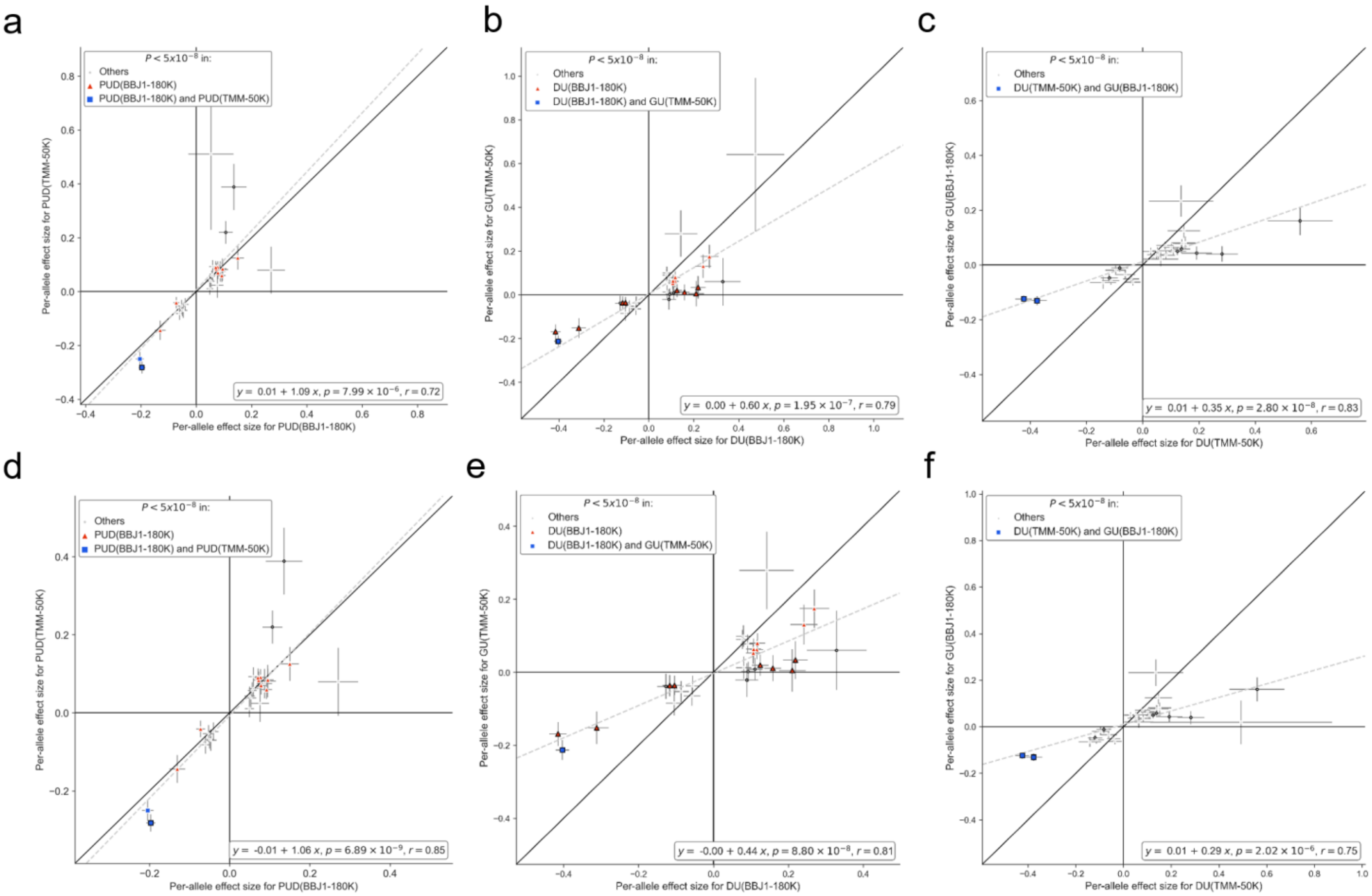

Supplementary Figure 21. Cross-cohort effect size comparison of distinct signals for DU and GU in East Asian ancestry individuals.

Per-allele effect size (log(OR)) comparison using summary statistics for PUD (**a,d**), DU (**b,e**), and GU (**c,f**) derived from GWASs in BBJ1-180K and TMM-50K. Lead variants and independent secondary signals associated with PUD or any subtypes in the EAS population were selected for comparison (GWAS P values are two-sided). The most significant associations were shown if overlapping variants exist (interval < 500 kb). For PUD, TMM-50K-derived statistics (N= 49,621) were compared with BBJ1-180K-derived statistics(N= 172,891; a,d). For DU and GU, TMM-50K-derived GU statistics(N= 47,536) were compared with BBJ1-180K-derived DU statistics (N= 160,345) (**b,e**), and TMM-50K-derived DU(N= 47,159) statistics were compared with BBJ1-180K-derived GU statistics(N= 167,591) (**c,f**). Data are presented as effect size estimates (log(OR)) +/- standard errors. Variants with significant heterogeneity (Cochran's Q test; two-sided $P_{het} < 0.05$) are denoted by black marker edges. The grey dashed line represents the fitted linear regression line with annotation at the bottom right (P values are derived from two-sided t-tests for the slopes). Marker colors denote the GWAS in which the significant variants were identified; Others, PUD. **a,** 28**, b,** 27**, c,** 27 available variants (existing in both datasets) are shown. **d,** 27**, e,** 26**, f,** 26 available variants with MAF>0.01 are shown. Source data are provided in Supplementary Table 29.

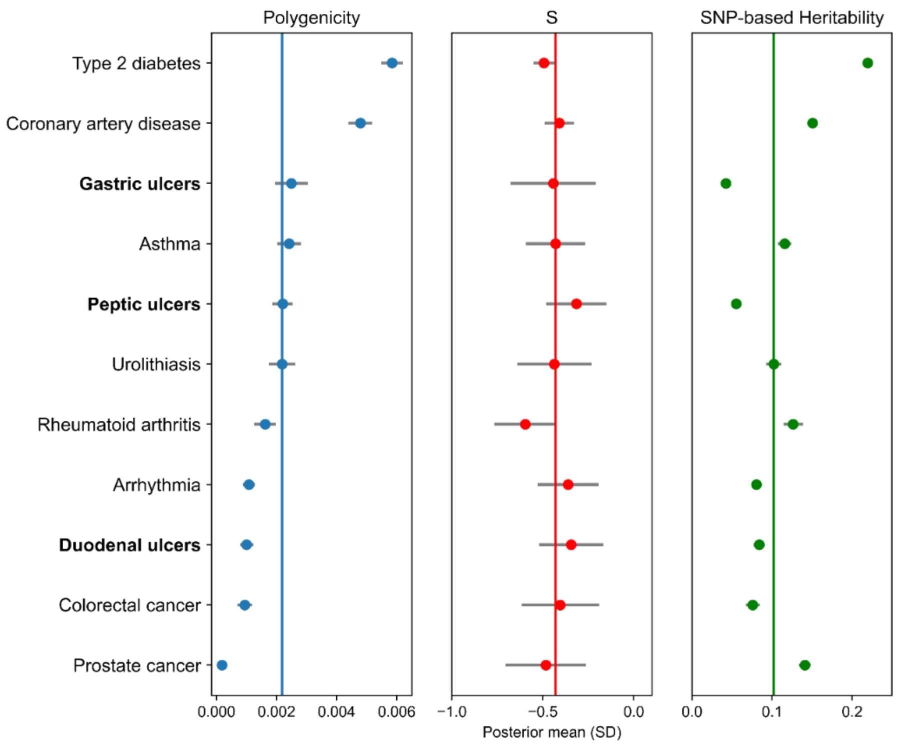

Supplementary Figure 22. Polygenicity estimation in East Asians using SBayesS.

Summary statistics for binary phenotypes from BBJ1[32] and meta-analysis of PUD and its subtypes in East Asians were used for SbayesS analysis. Only phenotypes that converged in the SbayesS model are shown. Phenotypes are ranked based on polygenicity estimates. SNP-based heritability estimates are on the liability scale. The vertical lines indicate the median estimates of the 11 phenotypes. Data are presented as posterior means +/- the standard deviations (SD) of the posterior means. Source data including sample size are provided in Supplementary Table 30.

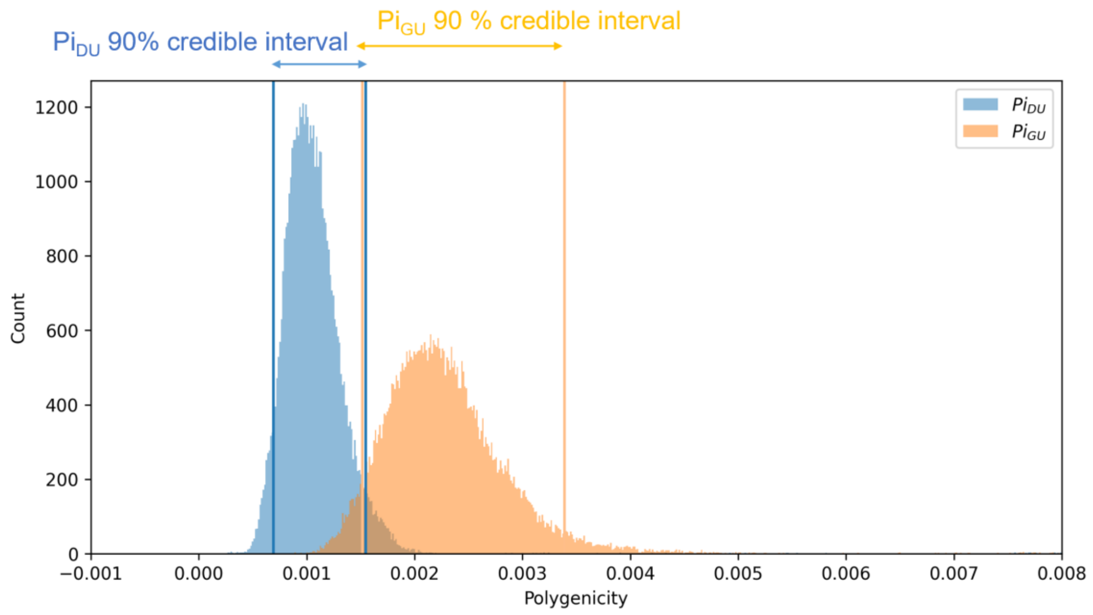

Supplementary Figure 23. Posterior distribution of polygenicity estimates for GU and DU in EAS using SbayesS.

The histogram shows the posterior distributions of the polygenicity for GU (Pi_{GU}) and DU (Pi_{DU}) estimated by SbayesS. Blue lines, the upper and lower bounds for the 90% credible interval for Pi_{DU}. Yellow lines, the upper and lower bounds for the 90% credible interval for Pi_{GU}.

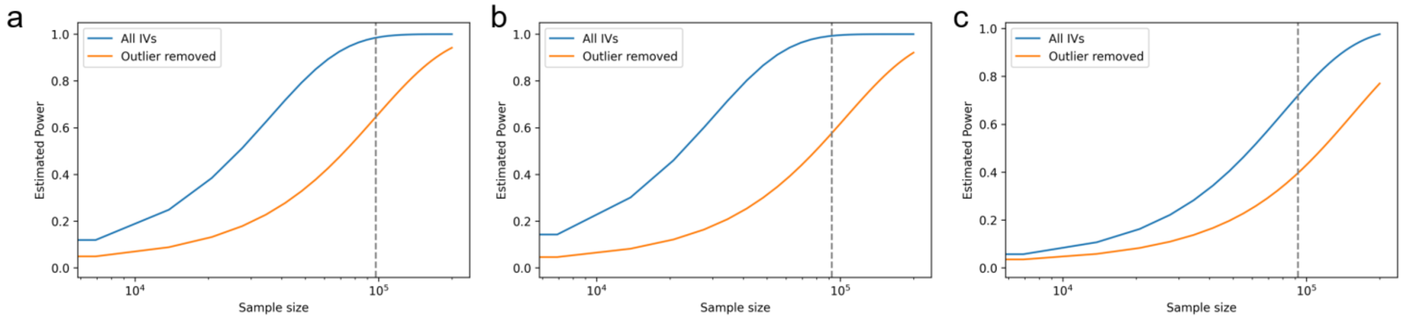

Supplementary Figure 24. Statistical power estimation for Mendelian randomization analysis.

The proportion of variance (r^2) explained by genetic instruments was approximated by the sum of the explained variance by the selected variants. Grey dashed lines represent the sample sizes. Significance, 0.0166; odds ratio, 1.2. **a**, Power estimation for the analysis of PUD; the ratio of cases to controls, 1:8.7; sample size, 97,523. **b**, Power estimation for the analysis of DU; the ratio of cases to controls, 1:18; sample size, 92,294. **c**, Power estimation for the analysis of GU; the ratio of cases to controls, 1:13; sample size, 94,056.

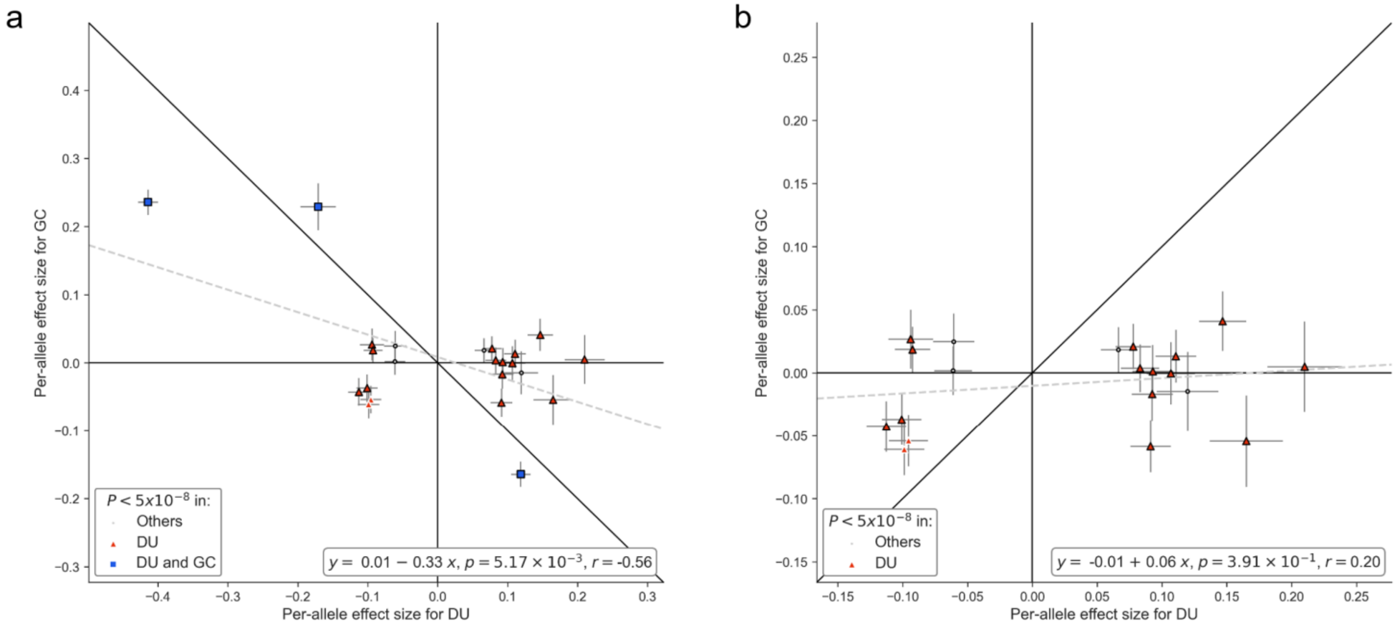

Supplementary Figure 25. Effect size comparison of distinct signals between PUD and GC in East Asian ancestry individuals.

Per-allele effect size (log10(OR)) comparison between DU and GC. Summary statistics for DU were obtained from EAS-specific meta-analysis (N= 252,639). Summary statistics for GC were obtained from previous GWAS conducted in BBJ1 (N= 195,745) (Methods). Lead variants and independent secondary signals associated with PUD or any subtypes in the EAS population were selected for comparison (GWAS P values are two-sided). Data are presented as effect size estimates (log(OR)) +/- standard errors. Variants with significant heterogeneity (Cochran's Q test; two-sided $P_{het} < 0.05$) are denoted by black marker edges. The grey dashed line represents the fitted linear regression line with annotation at the bottom right (P values are derived from two-sided t-tests for the slopes). The most significant associations are shown if overlapping variants exist (interval < 500 kb). **a**, 23 available variants (existing in both datasets) are shown. **b**, 20 variants (excluding lead variants at *EFNA1*, *PTGER4*, and *PSCA*) are shown. Source data including sample size are provided in Supplementary Table 35.

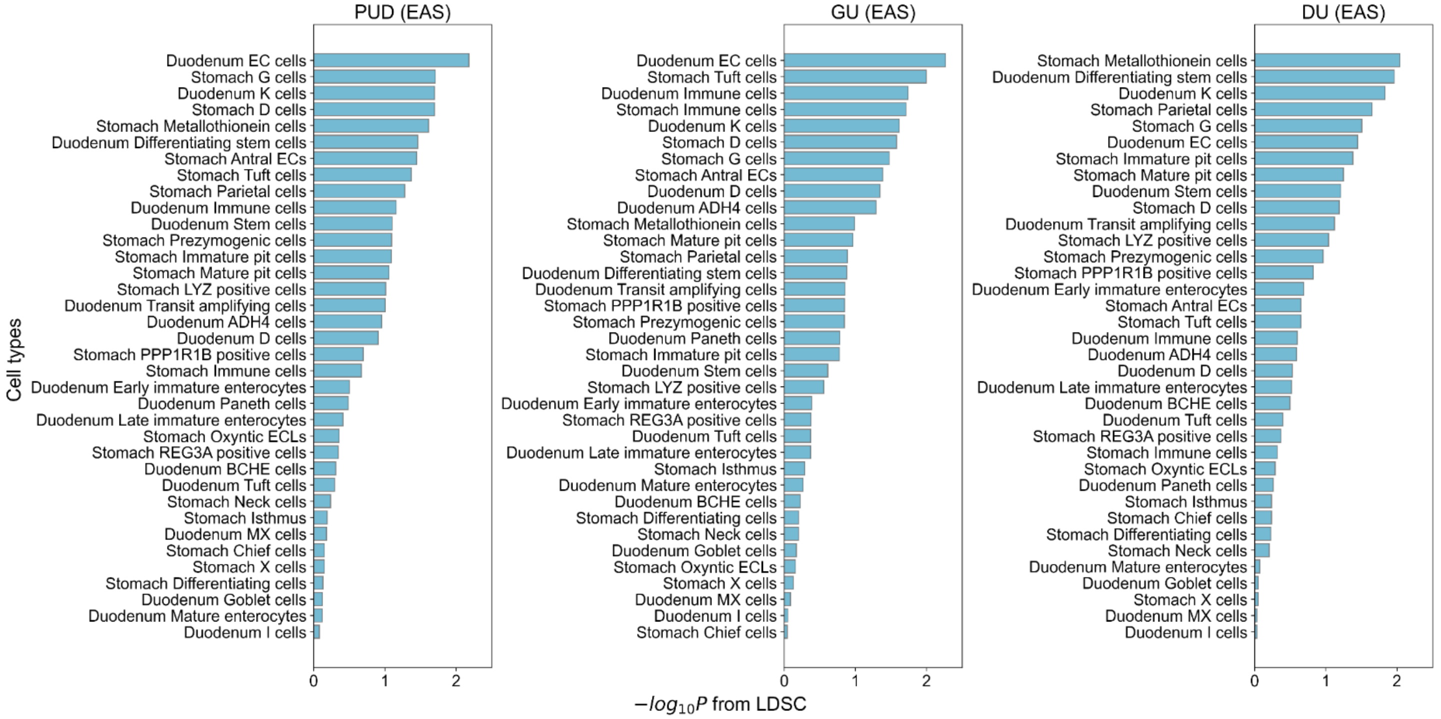

Supplementary Figure 26. Cell-type specificity analysis in East Asian ancestry individuals using LDSC.

Associations between PUD and cell types in the stomach and duodenum were analyzed using LDSC (testing for the enrichment of the 10% most specific genes in each cell type; **Methods**). East Asian-specific summary statistics were used in the analysis. X axis, –log10(P) (one-sided P values) derived from LDSC estimates. Color bars indicate whether the association is significant (Red, FDR<5%; Light blue, FDR>=5%).

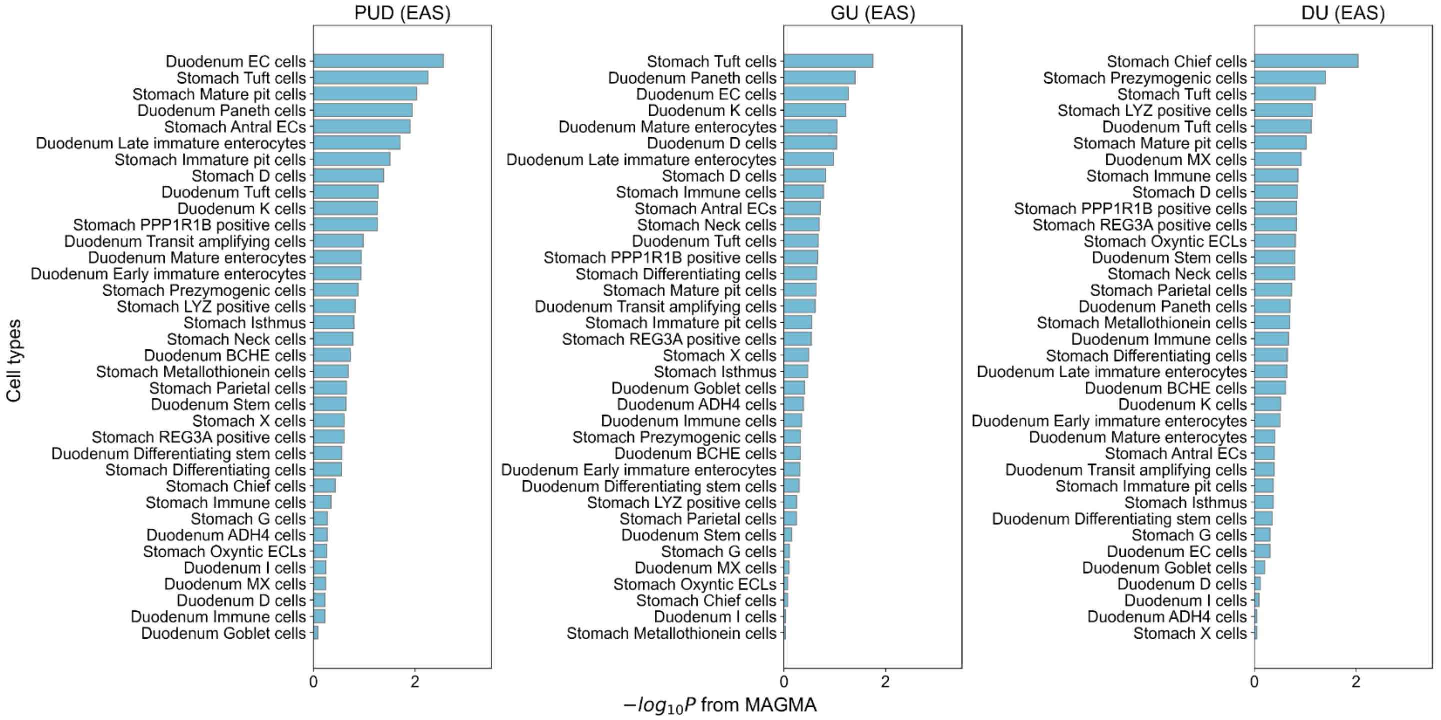

Supplementary Figure 27. Cell-type specificity analysis in East Asian ancestry individuals using MAGMA.

Associations between PUD and cell types in the stomach and duodenum were analyzed using MAGMA (testing for the enrichment of the 10% most specific genes in each cell type; **Methods**). East Asian-specific summary statistics were used in the analysis. X axis, –log10(P) (one-sided P values) derived from MAGMA estimates. Color bars indicate whether the association is significant (Red, FDR<5%; Light blue, FDR>=5%).

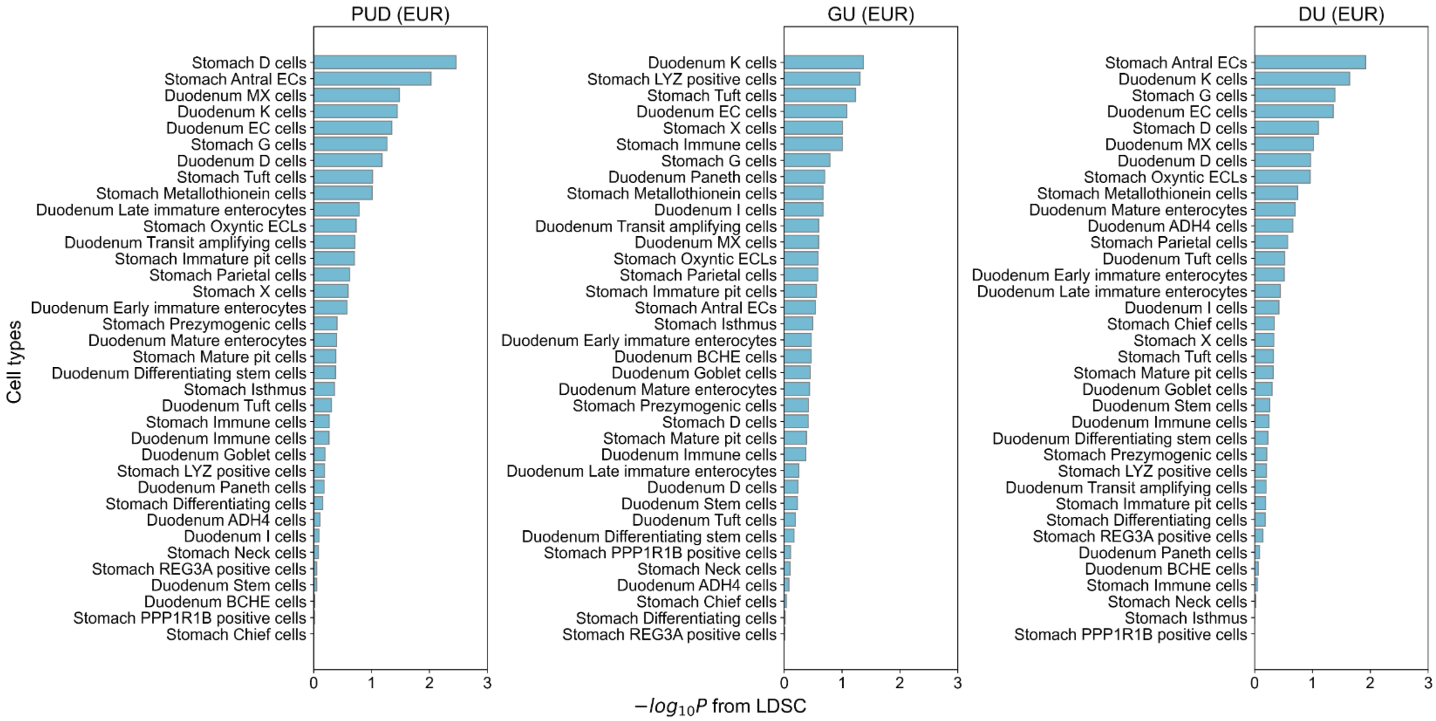

Supplementary Figure 28. Cell-type specificity analysis in European ancestry individuals using LDSC.

Associations between PUD and cell types in the stomach and duodenum were analyzed using LDSC (testing for the enrichment of the 10% most specific genes in each cell type; **Methods**). European-specific summary statistics were used in the analysis. X axis, –log10(P) (one-sided P values) derived from LDSC estimates. Color bars indicate whether the association is significant (Red, FDR<5%; Light blue, FDR>=5%).

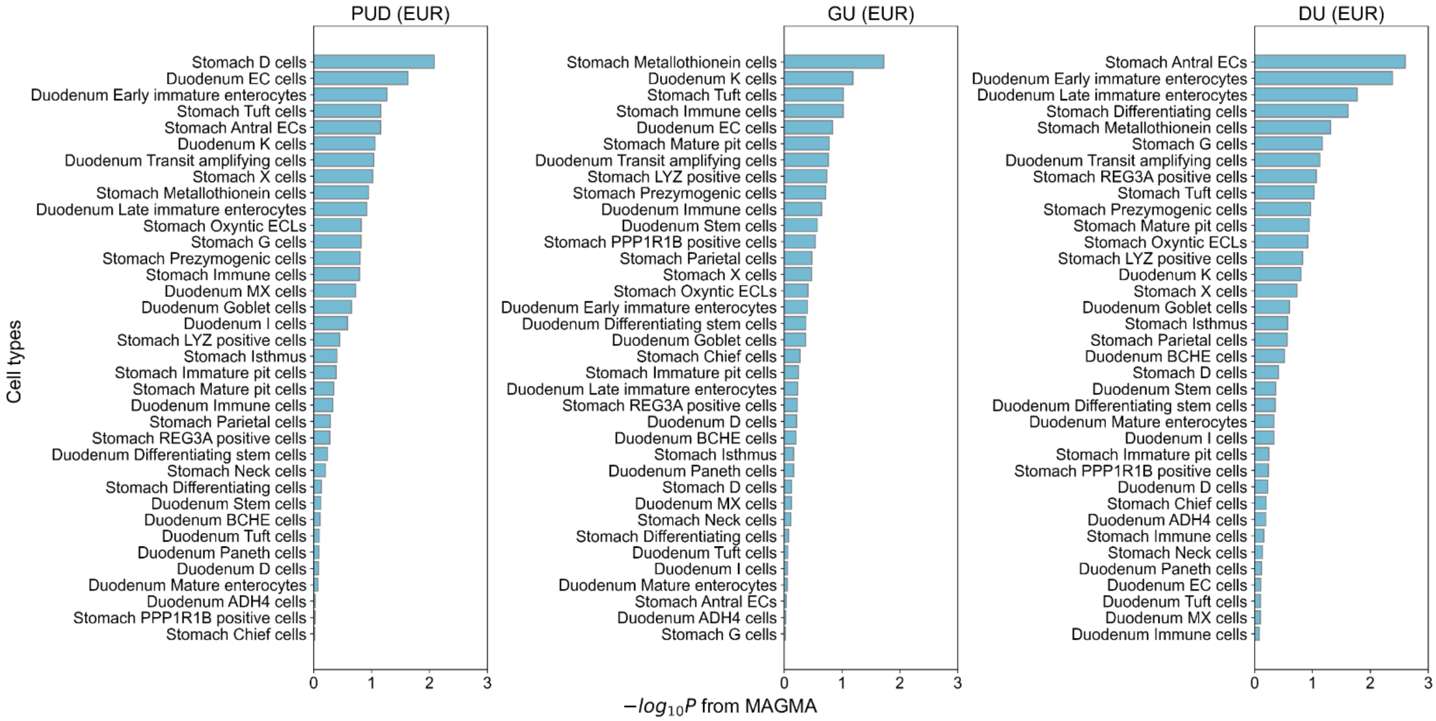

Supplementary Figure 29. Cell-type specificity analysis in European ancestry individuals using MAGMA.

Associations between PUD and cell types in the stomach and duodenum were analyzed using MAGMA (testing for the enrichment of the 10% most specific genes in each cell type; **Methods**). European-specific summary statistics were used in the analysis. X axis, –

log10(P) (one-sided P values) derived from MAGMA estimates. Color bars indicate whether the association is significant (Red, FDR<5%; Light blue, FDR>=5%).

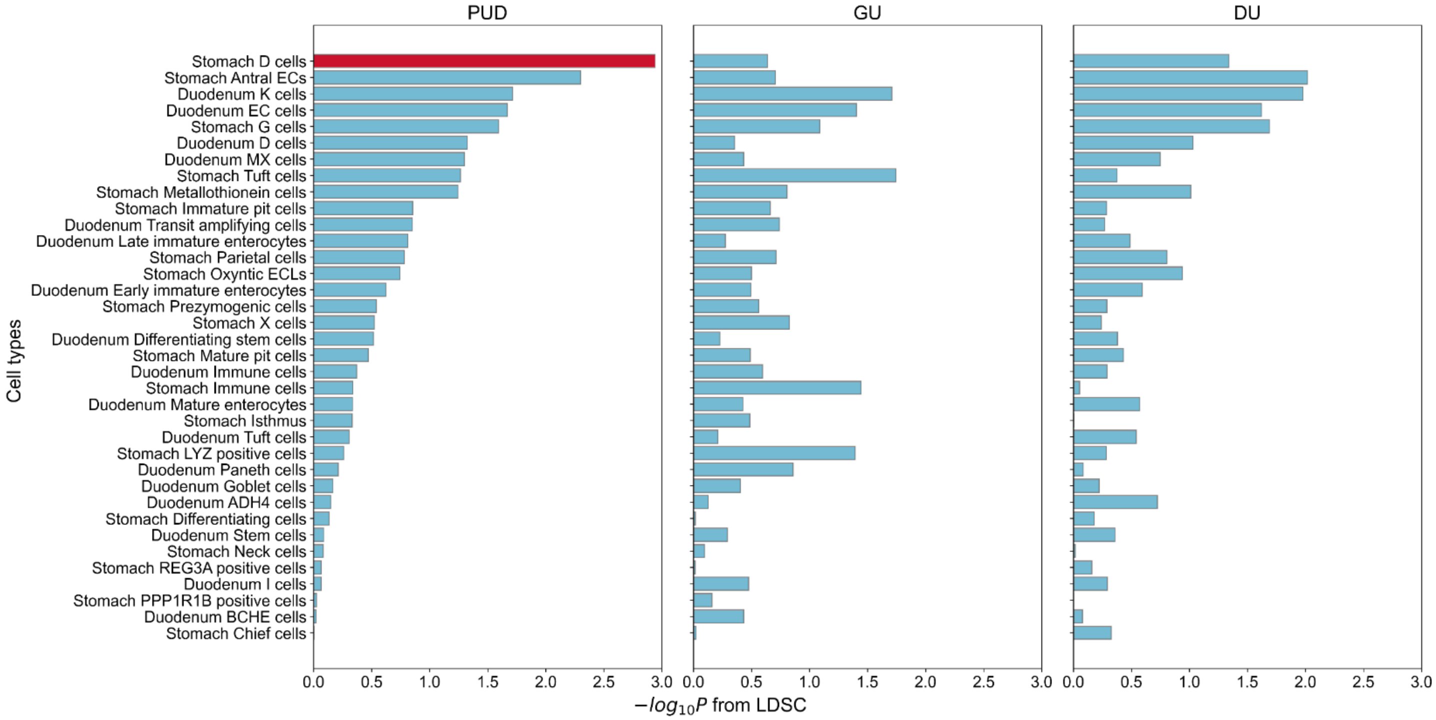

Supplementary Figure 30. Cross-ancestry meta-analysis of cell-type specificity using LDSC.

Associations between PUD and cell types in the stomach and duodenum were analyzed using LDSC (testing for the enrichment of the 10% most specific genes in each cell type). Inverse variance weighted meta-analysis was performed combining statistics from EAS and EUR ancestries. X axis, –log10(P) (one-sided P values) derived from meta-analyzed estimates. Color bars indicate whether the enrichment is significant (Red, FDR<5%; Light blue, FDR>=5%) in meta-analyzed results.

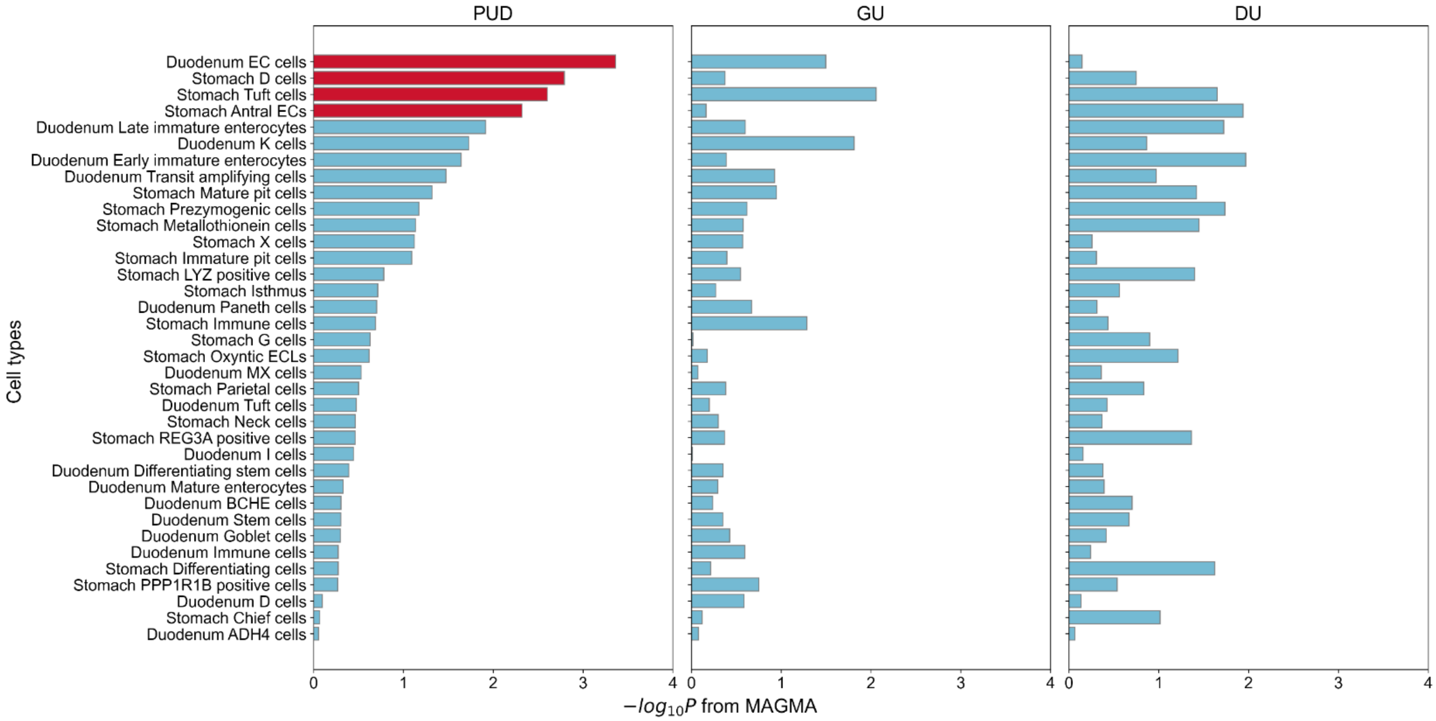

Supplementary Figure 31. Cross-ancestry meta-analysis of cell-type specificity using MAGMA.

Associations between PUD and cell types in the stomach and duodenum were analyzed using MAGMA (testing for the enrichment of the 10% most specific genes in each cell type). Inverse variance weighted meta-analysis was performed combining statistics from

EAS and EUR ancestries. X axis, –log10(P) (one-sided P values) derived from meta-analyzed estimates. Color bars indicate whether the enrichment is significant (Red, FDR<5%; Light blue, FDR>=5%) in meta-analyzed results.

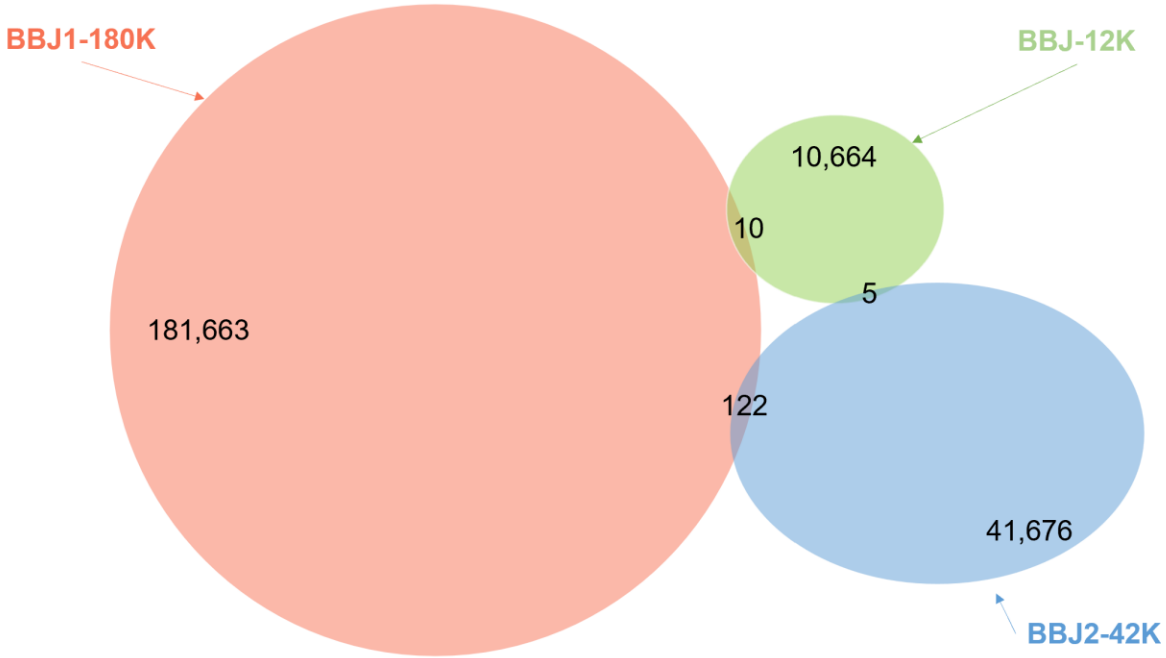

Supplementary Figure 32. Venn Plot of the potential sample overlap within Biobank Japan cohorts.

Sample overlap was estimated by IBD sharing.

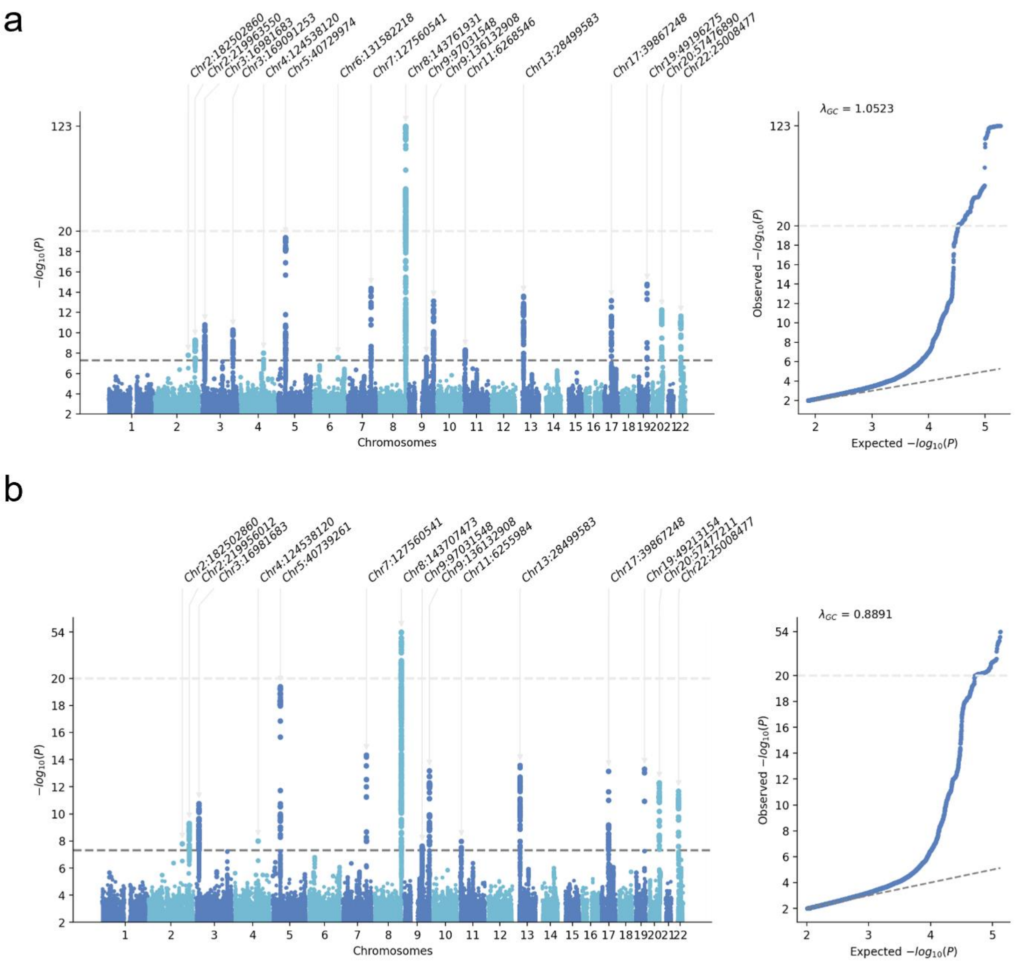

Supplementary Figure 33. Manhattan plots and Q-Q plots for EAS-specific meta-analysis of PUD using different models.

For variants above the top light grey dashed line (–log10(P) >20) (two-sided P values), values are rescaled. Lead variants are annotated with the nearest gene name. Variants are plotted against GRCh37 (hg19). The bottom dark grey dashed line indicates the genome-wide significance threshold (P < 5.0×10^{-8}). Variants with –log10(P) < 2 are omitted. **a**, Manhattan plot and Q-Q plot for the EAS-specific fixed-effect meta-analysis of PUD using METAL. **b**, Manhattan plot and Q-Q plot for the EAS-specific random-effects meta-analysis of PUD using GWAMA.

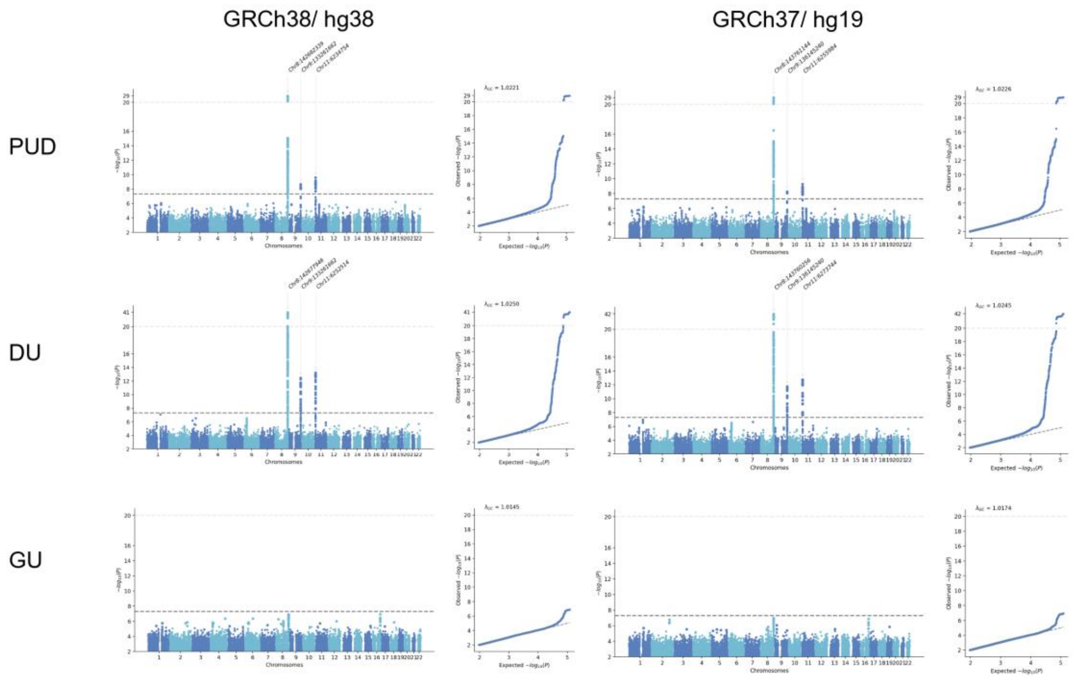

Supplementary Figure 34. Manhattan plots and Q-Q plots for cross-ancestry meta-analyses based on different versions of reference genome.

GWASs of PUD, DU, and GU conducted in BBJ1-12K, BBJ2-42K, and FinnGen were meta-analyzed (Methods). Variants existing in at least two cohorts are shown. For variants above the top light grey dashed line (–log10(P) >20) (two-sided P values), values are rescaled. The bottom dark grey dashed line indicates the genome-wide significance threshold ($P < 5.0 \times 10^{-8}$). Left panels, GRCh38-based datasets were used; variants are plotted against GRCh38 (hg38). Right panels, GRCh37-based datasets were used; variants are plotted against GRCh37 (hg19).

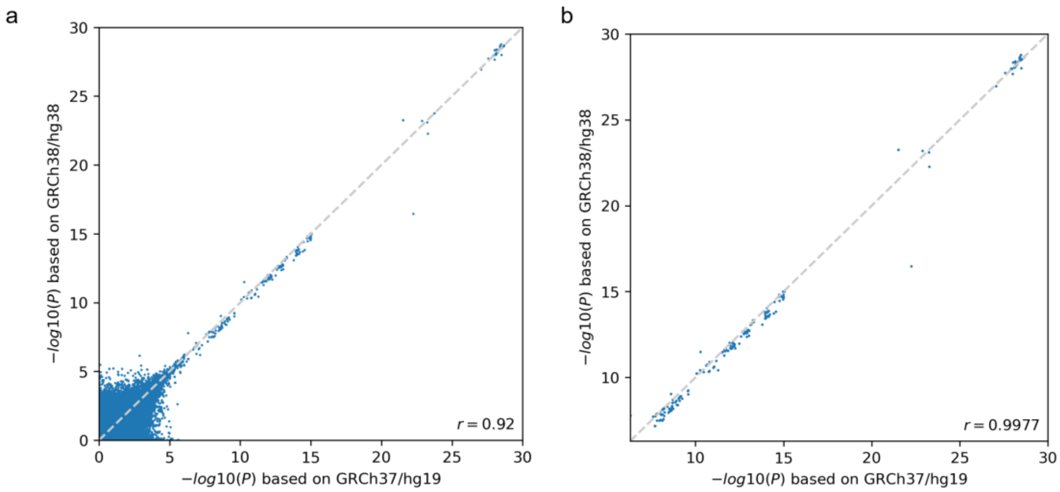

Supplementary Figure 35. Comparison of -log10(P) for SNPs in GWASs based on different versions of reference genome.

a, comparison of all shared SNPs between the GRCh37-based dataset and the GRCh38-based dataset (two-sided P values). The grey dashed line, the 45-degree line. Pearson correlation coefficient r is shown in the bottom right corner. **b**, comparison of SNPs reaching the genome-wide significance threshold ($P < 5.0 \times 10^{-8}$) (two-sided P values).

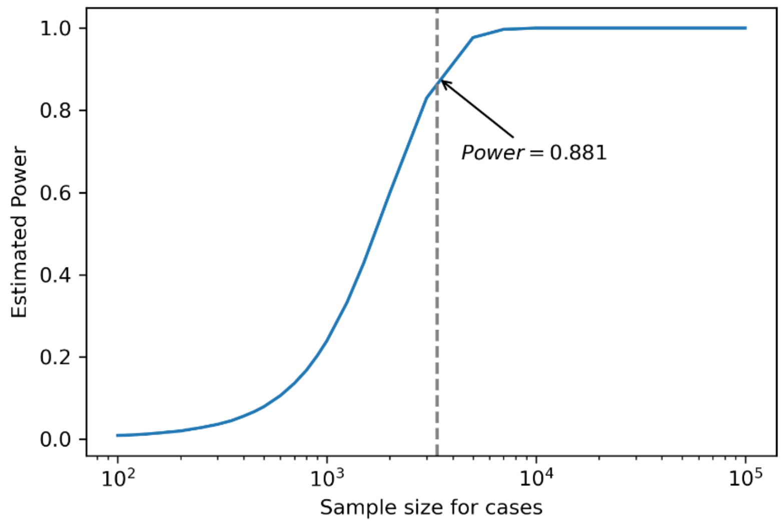

Supplementary Figure 36. Power analysis for GWAS of HP-negative PUD in TMM-50K.

Statistical power was estimated using GAS Power Calculator (https://csg.sph.umich.edu/abecasis/gas_power_calculator/). The dashed line represents the number of cases ($N_{case,\ HP\text{-}negative}$ = 3,372) used for GWAS of PUD in HP-negative individuals from TMM-50K. Other settings: the number of controls, 26,432; significance level, 0.0016; disease model, additive; prevalence, 0.109; disease allele frequency, 0.093; genotype relative risk, 1.189.

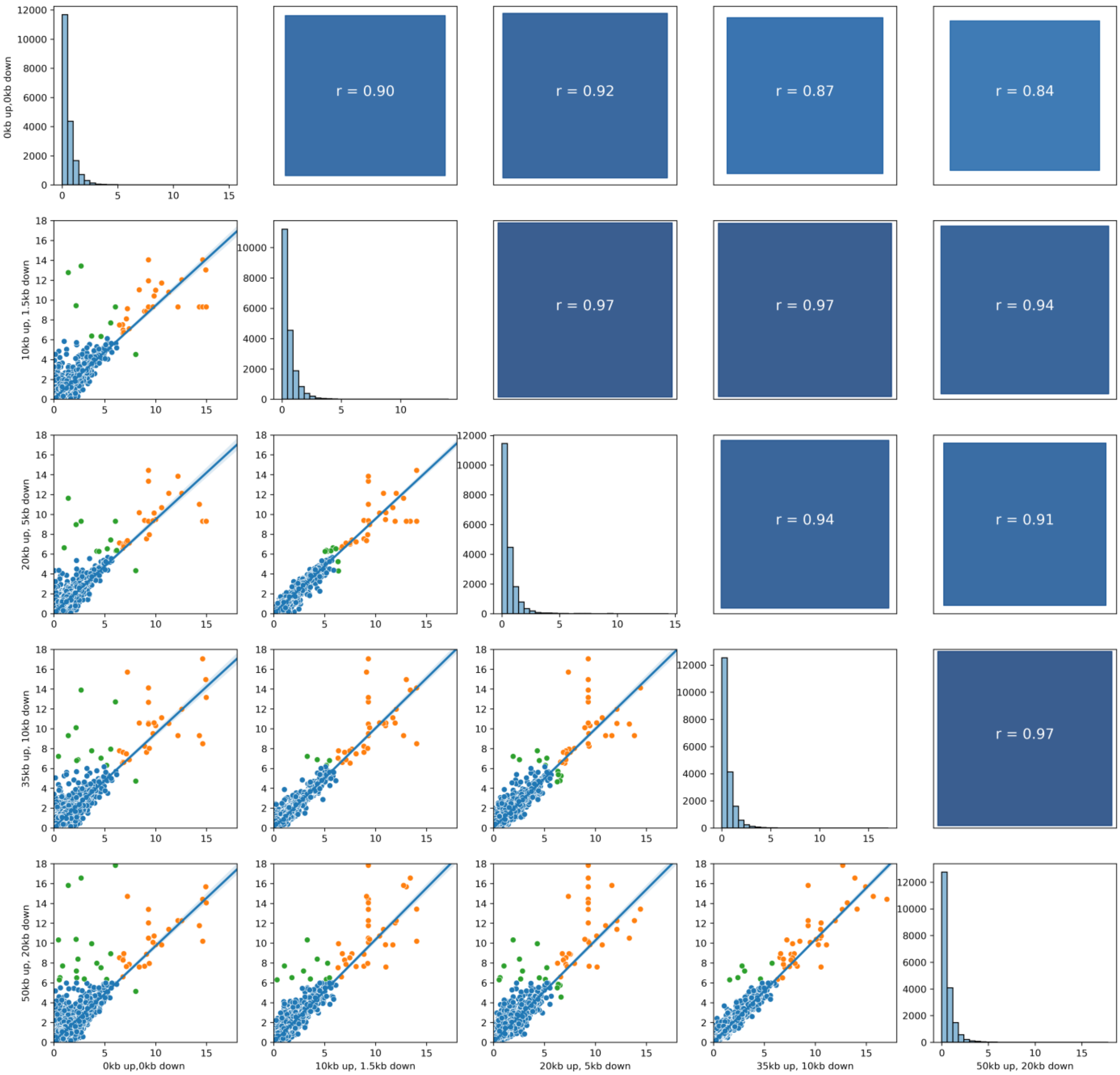

Supplementary Figure 37. Comparison of MAGMA results using different window sizes around genes for gene-based analysis.

Comparison of -log10(P) (one-sided P values) values obtained by gene-based tests for PUD in EAS using different window sizes for MAGMA (implemented in FUMA). Blue marker, P value after Bonferroni correction > 0.05 for both window sizes; green marker, P value after Bonferroni correction < 0.05 for only one of the two window sizes; yellow marker, P value after Bonferroni correction < 0.05 for both window sizes. Blue line, the linear regression line with confidence interval. The sizes of squares in the upper right are proportional to the Pearson correlation coefficient r.

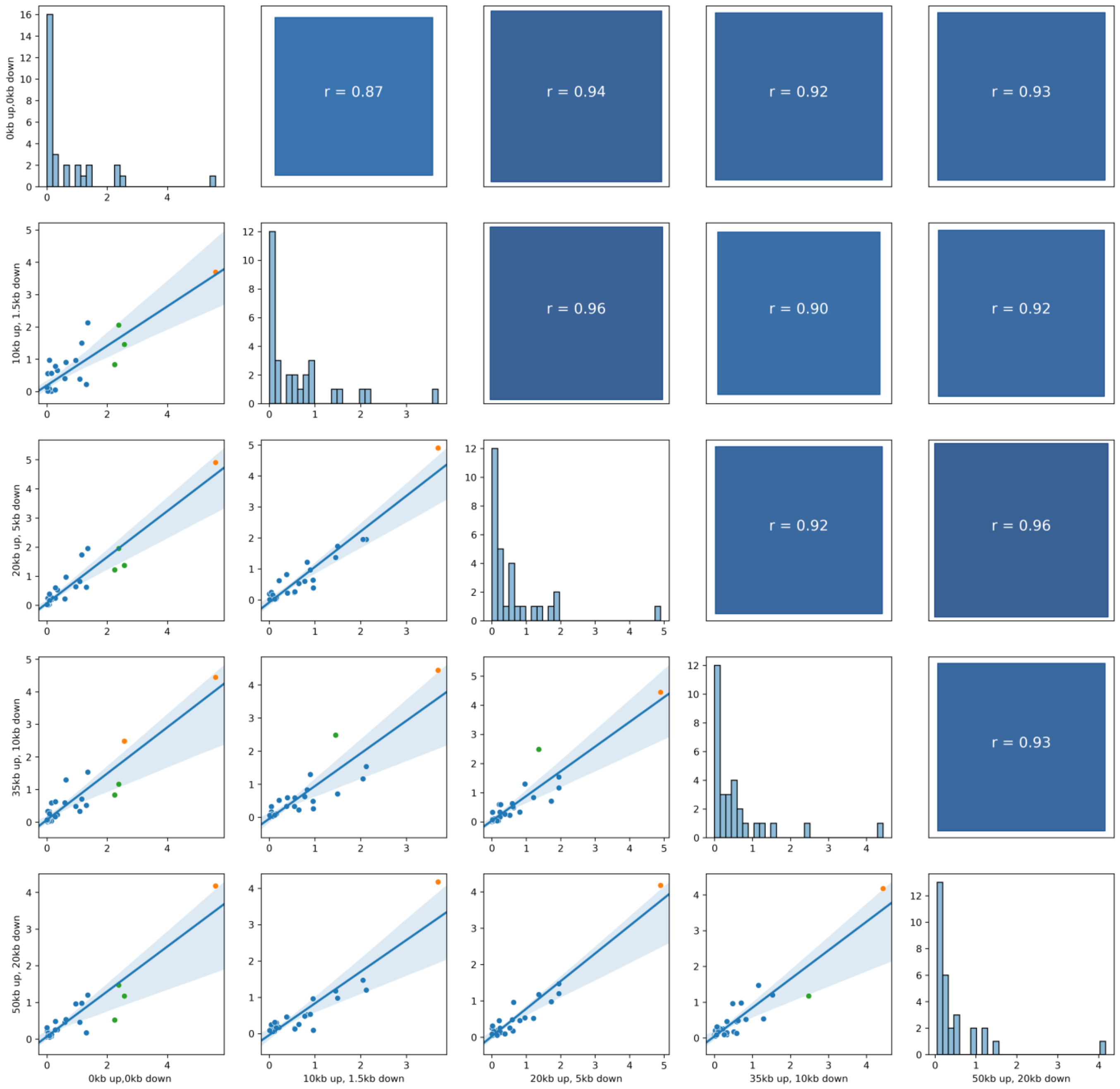

Supplementary Figure 38. Comparison of MAGMA results using different window sizes around genes for tissue-specificity analysis.

Comparison of -log10(P) (one-sided P values) values obtained by tissue-specificity analysis for PUD in EAS using different window sizes for MAGMA (implemented in FUMA). Blue marker, FDR > 5% for both window sizes; green marker, FDR < 5% for only one of the two window sizes; yellow marker, FDR < 5% for both window sizes. Blue line, the linear regression line with confidence interval. The sizes of squares in the upper right are proportional to the Pearson correlation coefficient r.

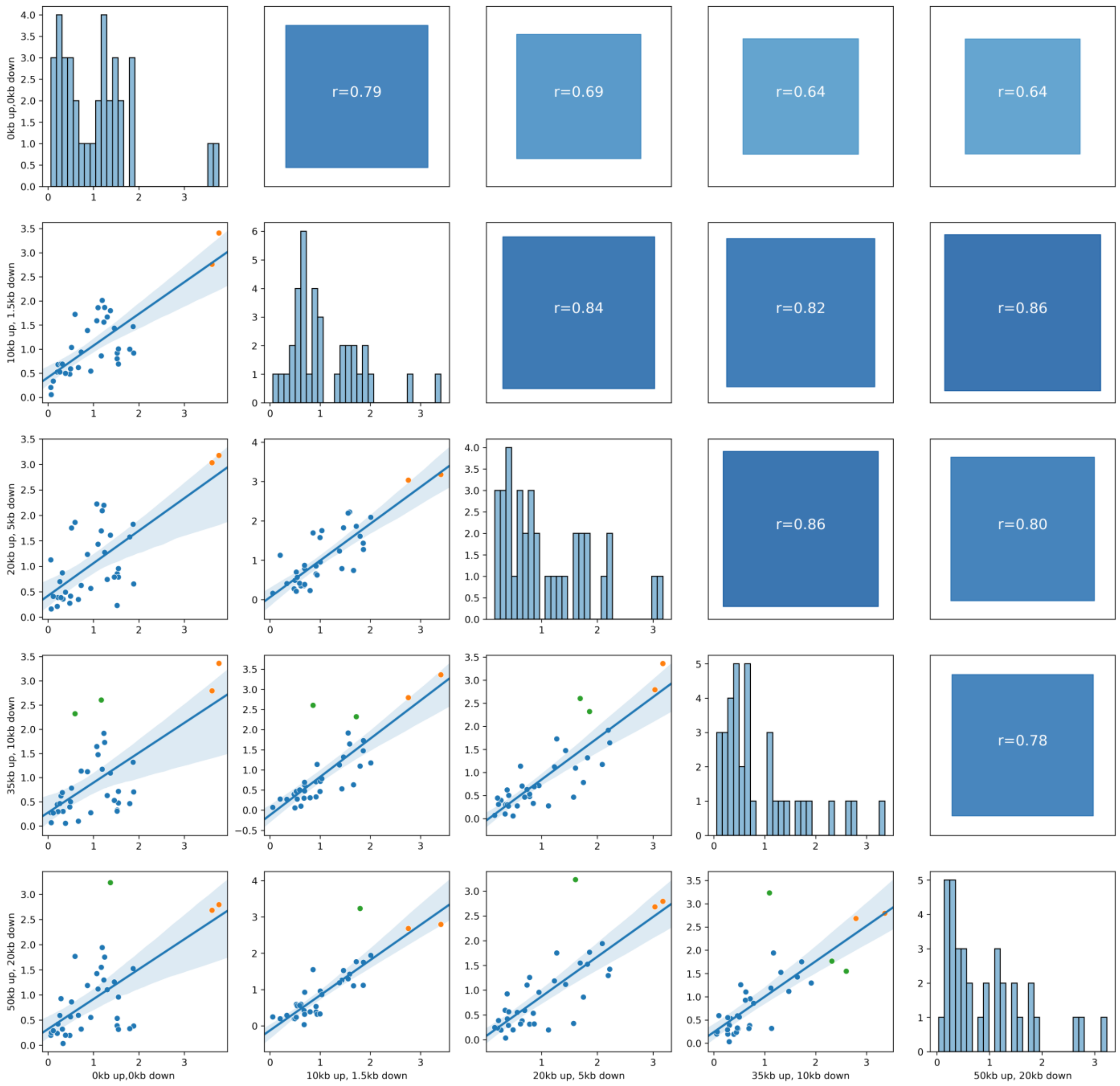

Supplementary Figure 39. Comparison of MAGMA results using different window sizes around genes for cell-type-specificity analysis.

Comparison of meta-analyzed -log10(P) (one-sided P values) values obtained by cell-type-specificity analysis for PUD using different window sizes. Blue marker, FDR > 5% for both window sizes; green marker, FDR < 5% for only one of the two window sizes; yellow marker, FDR < 5% for both window sizes. Blue line, the linear regression line with confidence interval. The sizes of squares in the upper right are proportional to the Pearson correlation coefficient r.

Members of participating consortia

BioBank Japan

Koichi Matsuda[6], Yuji Yamanashi[7], Yoichi Furukawa[8], Takayuki Morisaki[4,6], Yoshinori Murakami[4], Yoichiro Kamatani[1], Kaori Muto[5], Akiko Nagai[5], Wataru Obara[9], Ken Yamaji[10], Kazuhisa Takahashi[11], Satoshi Asai[12], Yasuo Takahashi[13], Takao Suzuki[14], Nobuaki Sinozaki[14], Hiroki Yamaguchi[15], Shiro Minami[16], Shigeo Murayama[17], Kozo Yoshimori[18], Satoshi Nagayama[19], Daisuke Obata[20], Masahiko Higashiyama[21], Akihide Masumoto[22] & Yukihiro Koretsune[23]

[1] Laboratory of Complex Trait Genomics, Graduate School of Frontier Sciences, The University of Tokyo, Tokyo, Japan
[4] Division of Molecular Pathology, Institute of Medical Science, The University of Tokyo, Tokyo, Japan
[5] Department of Public Policy, Institute of Medical Sciences, The University of Tokyo, Tokyo, Japan
[6] Laboratory of Clinical Genome Sequencing, Graduate School of Frontier Sciences, The University of Tokyo, Tokyo, Japan
[7] Division of Genetics, The Institute of Medical Science, The University of Tokyo, Tokyo, Japan.
[8] Division of Clinical Genome Research, Institute of Medical Science, The University of Tokyo, Tokyo, Japan.
[9] Department of Urology, Iwate Medical University, Iwate, Japan.
[10] Department of Internal Medicine and Rheumatology, Juntendo University Graduate School of Medicine, Tokyo, Japan.
[11] Department of Respiratory Medicine, Juntendo University Graduate School of Medicine, Tokyo, Japan.
[12] Division of Pharmacology, Department of Biomedical Science, Nihon University School of Medicine, Tokyo, Japan.
[13] Division of Genomic Epidemiology and Clinical Trials, Clinical Trials Research Center, Nihon University School of Medicine, Tokyo, Japan.
[14] Tokushukai Group, Tokyo, Japan.
[15] Departmentof Hematology, Nippon Medical School, Tokyo, Japan.
[16] Department of Bioregulation, Nippon Medical School, Kawasaki, Japan.
[17] Tokyo Metropolitan Geriatric Hospital and Institute of Gerontology, Tokyo, Japan.
[18] Fukujuji Hospital, Japan Anti-Tuberculosis Association, Tokyo, Japan.
[19] The Cancer Institute Hospital of the Japanese Foundation for Cancer Research, Tokyo, Japan.
[20] Center for Clinical Research and Advanced Medicine, Shiga University of Medical Science, Shiga, Japan.
[21] Department of General Thoracic Surgery, Osaka International Cancer Institute, Osaka, Japan.
[22] IIZUKA HOSPITAL, Fukuoka, Japan.
[23] National Hospital Organization Osaka National Hospital, Osaka, Japan.

References

1. Sun, B. B. *et al.* Genetic regulation of the human plasma proteome in 54,306 UK Biobank participants. 2022.06.17.496443 Preprint at https://doi.org/10.1101/2022.06.17.496443 (2022).
2. Gudjonsson, A. *et al.* A genome-wide association study of serum proteins reveals shared loci with common diseases. *Nat Commun* **13**, 480 (2022).
3. Sun, B. B. *et al.* Genomic atlas of the human plasma proteome. *Nature* **558**, 73–79 (2018).
4. Ferkingstad, E. *et al.* Large-scale integration of the plasma proteome with genetics and disease. *Nat Genet* **53**, 1712–1721 (2021).
5. Pietzner, M. *et al.* Mapping the proteo-genomic convergence of human diseases. *Science* **374**, eabj1541 (2021).
6. Kanaji, T. *et al.* A Common Genetic Polymorphism (46 C to T Substitution) in the 5′-Untranslated Region of the Coagulation Factor XII Gene Is Associated With Low Translation Efficiency and Decrease in Plasma Factor XII Level. *Blood* **91**, 2010–2014 (1998).
7. Chattopadhyay, R., Sengupta, T. & Majumder, R. Inhibition of intrinsic Xase by protein S: a novel regulatory role of protein S independent of activated protein C. *Arterioscler Thromb Vasc Biol* **32**, 2387–2393 (2012).
8. Grover, S. P. & Mackman, N. Intrinsic Pathway of Coagulation and Thrombosis. *Arterioscler Thromb Vasc Biol* **39**, 331–338 (2019).
9. Li, L. F. *et al.* Cigarette smoking and gastrointestinal diseases: the causal relationship and underlying molecular mechanisms (review). *Int J Mol Med* **34**, 372–380 (2014).

10. Wannamethee, S. G. & Shaper, A. G. Cigarette smoking and serum liver enzymes: the role of alcohol and inflammation. *Ann Clin Biochem* **47**, 321–326 (2010).

11. Jang, E. S. *et al.* Effects of coffee, smoking, and alcohol on liver function tests: a comprehensive cross-sectional study. *BMC Gastroenterol* **12**, 145 (2012).

12. Tsuo, K. *et al.* Multi-ancestry meta-analysis of asthma identifies novel associations and highlights the value of increased power and diversity. *Cell Genom* **2**, 100212 (2022).

13. Rorsman, P. & Huising, M. O. The somatostatin-secreting pancreatic δ-cell in health and disease. *Nat Rev Endocrinol* **14**, 404–414 (2018).

14. Ampofo, E., Nalbach, L., Menger, M. D. & Laschke, M. W. Regulatory Mechanisms of Somatostatin Expression. *Int J Mol Sci* **21**, 4170 (2020).

15. Smith, S. B., Ee, H. C., Conners, J. R. & German, M. S. Paired-Homeodomain Transcription Factor PAX4 Acts as a Transcriptional Repressor in Early Pancreatic Development. *Molecular and Cellular Biology* **19**, 8272–8280 (1999).

16. Larsson, L. I., St-Onge, L., Hougaard, D. M., Sosa-Pineda, B. & Gruss, P. Pax 4 and 6 regulate gastrointestinal endocrine cell development. *Mech Dev* **79**, 153–159 (1998).

17. Beucher, A. *et al.* The homeodomain-containing transcription factors Arx and Pax4 control enteroendocrine subtype specification in mice. *PLoS One* **7**, e36449 (2012).

18. Goudet, G., Delhalle, S., Biemar, F., Martial, J. A. & Peers, B. Functional and cooperative interactions between the homeodomain PDX1, Pbx, and Prep1 factors on the somatostatin promoter. *J Biol Chem* **274**, 4067–4073 (1999).

19. Latorre, R., Sternini, C., De Giorgio, R. & Greenwood-Van Meerveld, B. Enteroendocrine cells: a review of their role in brain-gut communication. *Neurogastroenterol Motil* **28**, 620–630 (2016).

20. Hsu, C.-C. *et al.* Depression and the Risk of Peptic Ulcer Disease: A Nationwide Population-Based Study. *Medicine (Baltimore)* **94**, e2333 (2015).

21. Deding, U. *et al.* Perceived stress as a risk factor for peptic ulcers: a register-based cohort study. *BMC Gastroenterol* **16**, 140 (2016).

22. Wu, Y. *et al.* GWAS of peptic ulcer disease implicates Helicobacter pylori infection, other gastrointestinal disorders and depression. *Nat Commun* **12**, 1146 (2021).

23. Mägi, R. & Morris, A. P. GWAMA: software for genome-wide association meta-analysis. *BMC Bioinformatics* **11**, 288 (2010).

24. Nagai, A. *et al.* Overview of the BioBank Japan Project: Study design and profile. *J Epidemiol* **27**, S2–S8 (2017).

25. 1000 Genomes Project Consortium *et al.* A global reference for human genetic variation. *Nature* **526**, 68–74 (2015).

26. Hozawa, A. *et al.* Study Profile of the Tohoku Medical Megabank Community-Based Cohort Study. *J Epidemiol* **31**, 65–76 (2021).

27. Akiyama, M. *et al.* Characterizing rare and low-frequency height-associated variants in the Japanese population. *Nat Commun* **10**, 4393 (2019).

28. Tan, A., Abecasis, G. R. & Kang, H. M. Unified representation of genetic variants. *Bioinformatics* **31**, 2202–2204 (2015).

29. GTEx Consortium. The GTEx Consortium atlas of genetic regulatory effects across human tissues. *Science* **369**, 1318–1330 (2020).

30. Matoba, N. *et al.* GWAS of 165,084 Japanese individuals identified nine loci associated with dietary habits. *Nat Hum Behav* **4**, 308–316 (2020).

31. Kanai, M. *et al.* Genetic analysis of quantitative traits in the Japanese population links cell types to complex human diseases. *Nat Genet* **50**, 390–400 (2018).

32. Ishigaki, K. *et al.* Large-scale genome-wide association study in a Japanese population identifies novel susceptibility loci across different diseases. *Nat Genet* **52**, 669–679 (2020).

33. Sakaue, S. *et al.* A cross-population atlas of genetic associations for 220 human phenotypes. *Nat Genet* **53**, 1415–1424 (2021).
