# Supplementary material for: East Asian-specific and cross-ancestry genome-wide meta-analyses provide mechanistic insights into peptic ulcer disease
Source: Nat Genet. 2023 Nov 30;55(12):2129–38. doi: 10.1038/s41588-023-01569-7 (PMC10703676; doi:10.1038/s41588-023-01569-7)
Supplement: Supplementary file 2 — Reporting Summary [file 41588_2023_1569_MOESM2_ESM.pdf]

nature portfolio

Corresponding author(s): Yoichiro Kamatani
Last updated by author(s): Jun 21, 2023

Reporting Summary

Nature Portfolio wishes to improve the reproducibility of the work that we publish. This form provides structure for consistency and transparency in reporting. For further information on Nature Portfolio policies, see our Editorial Policies and the Editorial Policy Checklist.

Statistics

For all statistical analyses, confirm that the following items are present in the figure legend, table legend, main text, or Methods section.

n/a	Confirmed	
☐	☒	The exact sample size (*n*) for each experimental group/condition, given as a discrete number and unit of measurement
☐	☒	A statement on whether measurements were taken from distinct samples or whether the same sample was measured repeatedly
☐	☒	The statistical test(s) used AND whether they are one- or two-sided<br>*Only common tests should be described solely by name; describe more complex techniques in the Methods section.*
☐	☒	A description of all covariates tested
☐	☒	A description of any assumptions or corrections, such as tests of normality and adjustment for multiple comparisons
☐	☒	A full description of the statistical parameters including central tendency (e.g. means) or other basic estimates (e.g. regression coefficient) AND variation (e.g. standard deviation) or associated estimates of uncertainty (e.g. confidence intervals)
☐	☒	For null hypothesis testing, the test statistic (e.g. *F*, *t*, *r*) with confidence intervals, effect sizes, degrees of freedom and *P* value noted<br>*Give P values as exact values whenever suitable.*
☐	☒	For Bayesian analysis, information on the choice of priors and Markov chain Monte Carlo settings
☒	☐	For hierarchical and complex designs, identification of the appropriate level for tests and full reporting of outcomes
☐	☒	Estimates of effect sizes (e.g. Cohen's *d*, Pearson's *r*), indicating how they were calculated

Our web collection on statistics for biologists contains articles on many of the points above.

Software and code

Policy information about availability of computer code

Data collection	No software was used.
Data analysis	We used publicly available software for the analysis, including Plink (v1.9 and v2.0), Eagle2 (v2.4.1), Minimac4 (v1.0.2), SAIGE (v0.44), LDSC (v1.0.0), ANNOVAR (v2020-06-07), METAL (v2011-03-25), GWAMA(v2.2.2), UCSC LiftOver tool, MR-MEGA (v0.2), Popcorn (v1.0), Python(v3.8.8), R(v4.1.0), LocusZoom (v1.2), GCTA-COJO (v1.93.2), SuSiE (v0.11.92), LDstore (v2.0), metafor (v3.4), coloc (v5.1.0), GCTB (v2.0), PRScs (v2021-Jun-4), TwoSampleMR(0.5.6), MAGMA (v1.08), and FUMA (v1.3.8).

For manuscripts utilizing custom algorithms or software that are central to the research but not yet described in published literature, software must be made available to editors and reviewers. We strongly encourage code deposition in a community repository (e.g. GitHub). See the Nature Portfolio guidelines for submitting code & software for further information.

Data

Policy information about availability of data

All manuscripts must include a data availability statement. This statement should provide the following information, where applicable:

- Accession codes, unique identifiers, or web links for publicly available datasets
- A description of any restrictions on data availability
- For clinical datasets or third party data, please ensure that the statement adheres to our policy

Summary statistics for GWAS of PUD and PUD subtypes in BBJ1-180K, BBJ1-12K and BBJ2-42K, and EAS-specific and cross-ancestry meta-analysis summary statistics

are available at National Bioscience Database Center (NBDC, https://humandbs.biosciencedbc.jp/) Human Database (research ID: hum0311) and Japanese ENcyclopedia of GEnetic associations by Riken (JENGER, http://jenger.riken.jp/result; Case-control GWAS #135 - #155). EAS-specific and cross-ancestry meta-analysis summary statistics are additionally deposited to EBI GWAS Catalog (Study Accession ID: GCST90270926 - GCST90270932). Summary statistics derived from TMM-50K (GWAS of PUD and PUD subtypes; HP-stratified analysis) will be available at the Japanese Multi Omics Reference Panel (jMorp, https://jmorp.megabank.tohoku.ac.jp; ID: TGA000011). Genotype data for BBJ were deposited at NBDC Human Database (BBJ1-180K, research ID:hum0014; BBJ1-12K and BBJ2-42K, research ID:hum0311). Summary statistics for European individuals were obtained from FinnGen release 6 (https://www.finngen.fi/en/access_results), and UKB datasets (https://cnsgenomics.com/content/data and https://pheweb.org/UKB-SAIGE/). Summary statistics for other traits in BBJ were obtained from Japanese ENcyclopedia of GEnetic associations by Riken (JENGER, http://jenger.riken.jp/), BioBank Japan PheWeb (https://pheweb.jp/phenotypes) and NBDC (Research ID: hum0014).

Human research participants

Policy information about studies involving human research participants and Sex and Gender in Research.

Reporting on sex and gender	We performed sex (biological attribute)-stratified analysis in BBJ1-180K. In this analysis, sex was assigned when the self-reporting sex matched the sex imputed from X chromosome inbreeding coefficients. In total, 78,211 males and 74,967 females in BBJ1-180K were included in the sex-stratified analysis. Detailed descriptions and results were reported in Supplementary Table 1 and Supplementary Table 3.
Population characteristics	BioBank Japan Project (BBJ) is a hospital-based study that recruited approximately 200,000 participants (mainly of Japanese ancestry) from 2003 to 2007 and additionally recruited approximately 67,000 participants from 2013 to 2017.<br>The Tohoku Medical Megabank Project (TMM) involves two prospective cohort studies, one is a population-based adult cohort study of 80,000 participants, and the other is a birth and three-generation cohort study of 70,000 participants. In this study, we used the data from a part of the population-based cohort study.<br>The population characteristics are provided in detail in Supplementary Table 1.
Recruitment	BioBank Japan Project (BBJ) recruited participants at 66 hospitals (BBJ1) and 52 hospitals (BBJ2) with the support from 12 medical institutions. In each recruitment period, patients were enrolled who were diagnosed as one of 47 common diseases between 2003 and 2007 (BBJ1), or one of 38 diseases (mostly overlapped) between 2013 and 2017 (BBJ2). (https://biobankjp.org/en/index.html#01)<br>In the population-based adult cohort study of the Tohoku Medical Megabank Project (TMM), participants were recruited on a voluntary basis through the specific health checkup conducted by municipalities and also at the seven "Community Support Centers" or five "Satellites" in Miyagi and Iwate Prefectures in Japan, from 2013 to 2016.
Ethics oversight	All the participants from BBJ and TMM provided written informed consent. The research project was approved by the ethics committees at the Institute of Medical Science, the University of Tokyo (application number 29-74-A0215), and Iwate Tohoku Medical Megabank Organization, Iwate Medical University (application number HG H25-2).

Note that full information on the approval of the study protocol must also be provided in the manuscript.

Field-specific reporting

Please select the one below that is the best fit for your research. If you are not sure, read the appropriate sections before making your selection.

☒ Life sciences ☐ Behavioural & social sciences ☐ Ecological, evolutionary & environmental sciences

For a reference copy of the document with all sections, see nature.com/documents/nr-reporting-summary-flat.pdf

Life sciences study design

All studies must disclose on these points even when the disclosure is negative.

Sample size	The sample size of GWASs in this study is summarized in Supplementary Table 1 and 7. We did not perform sample size calculation, and the sample size is determined by the maximum number of individuals in each cohort who passed sample QC, which is expected to increase the statistical power. To further increase the sample size, we obtained publicly available summary statistics from UK Biobank and Finngen studies, and performed a cross-ancestry meta-analysis.
Data exclusions	All samples were selected based on sample QC criteria for each cohort. Briefly, samples with age<18, non-EAS ancestry, or low call rate were excluded for BBJ1-180K, BBJ1-12K, BBJ2-42K and TMM-50K. Additionally, samples with amyotrophic lateral sclerosis in BBJ1-12K due to its comparatively high proportion. The detailed description is summarized in the Method section. All association analysis was performed using the dataset after QC.
Replication	Replication for the discovery GWAS in EAS was conducted in individuals from three independent studies, namely BBJ1-12K, BBJ2-40K, and TMM-50K. We confirmed relatively high replicability (among the nine novel lead variants associated with PUD or subtypes, four were nominally associated with PUD or its subtypes in the same direction in at least two replication datasets and five novel loci were replicated in the population-based TMM dataset). We further conducted the cross-ancestry comparison of the effect sizes.
Randomization	We did not apply randomization in this study since this is a genotype-phenotype association study. All the samples passed the sample QC were included in the analysis. For SNV association tests performed in this study, age, sex and top 10 principal components were adjusted in

	the regression.
Blinding	We did not apply blinding in this study since this is a genotype-phenotype association study. No intervention was involved in this study.

Reporting for specific materials, systems and methods

We require information from authors about some types of materials, experimental systems and methods used in many studies. Here, indicate whether each material, system or method listed is relevant to your study. If you are not sure if a list item applies to your research, read the appropriate section before selecting a response.

Materials & experimental systems

n/a	Involved in the study
☒	☐ Antibodies
☒	☐ Eukaryotic cell lines
☒	☐ Palaeontology and archaeology
☒	☐ Animals and other organisms
☒	☐ Clinical data
☒	☐ Dual use research of concern

Methods

n/a	Involved in the study
☒	☐ ChIP-seq
☒	☐ Flow cytometry
☒	☐ MRI-based neuroimaging
